# Reversible mechanical protection: building a 3D “suit” around a T-shaped benzimidazole axle†
†Electronic supplementary information (ESI) available: Synthetic details and full characterisation of all new compounds. CCDC 1533271 and 1533272. For ESI and crystallographic data in CIF or other electronic format see DOI: 10.1039/c7sc00790f



**DOI:** 10.1039/c7sc00790f

**Published:** 2017-03-28

**Authors:** Kelong Zhu, Giorgio Baggi, V. Nicholas Vukotic, Stephen J. Loeb

**Affiliations:** a School of Chemistry , Sun Yat-Sen University , Guangzhou , 510275 , P. R. China . Email: zhukelong@mail.sysu.edu.cn; b Department of Chemistry and Biochemistry , University of Windsor , Windsor , Ontario N9B 3P4 , Canada . Email: loeb@uwindsor.ca

## Abstract

The T-shaped benzimidazolium/crown ether recognition motif was used to prepare suit[1]anes. These novel mechanically interlocked molecules (MIMs) were fully characterized by ^1^H and ^13^C NMR spectroscopy, single-crystal X-ray diffraction, UV-vis absorption and fluorescence spectroscopy. By conversion to a suit[1]ane, a simple benzimidazole was shown to be protected from deprotonation by strong base. Moreover, it was demonstrated that this unique three-dimensional encapsulation can be made reversible, thus introducing the concept of “reversible mechanical protection”; a protecting methodology that may have potential applications in synthetic organic chemistry and the design of molecular machinery.

## Abstract 2

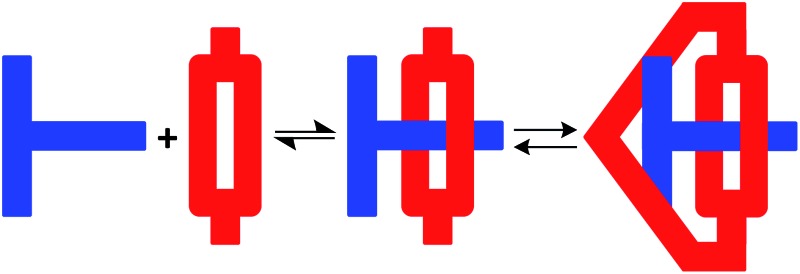
A benzimidazole molecule can be protected from deprotonation with strong base by converting into a suit[1]ane – a type of 3D mechanically interlocked molecule. Utilising a combination of ring-closing and ring-opening metathesis, the wearing of the protective “suit” can be made reversible.

## Introduction

Mechanically interlocked molecules (MIMs) are assemblies of molecular components or entanglements that cannot be separated without breaking a covalent bond.^1^ The combination of template-directed synthesis^2^ and mechanically interlocked topologies^3^ has allowed the synthesis of a variety of MIMs such as rotaxanes,^4^ catenanes,^5^ daisy chains,^6^ knots^7^ and Borromean rings.^8^ The dynamic nature of MIMs has also been exploited as the basis for developing artificial molecular machines by manipulating the relative positions of their constituent components, especially for rotaxanes and catenanes.^9^

An underappreciated consequence of mechanically interlocking two molecular components is that the permanence of their intimacy can dramatically affect the chemical properties of the individual components.^10^ Indeed, it is possible to not only stabilize vulnerable molecules,^11^ but also develop functional materials such as ‘molecular wires’ by virtue of the interpenetrated structure of rotaxanes.^12^ In a previous study, we showed how this protecting methodology could be used to alter reactivity, by wrapping a polyether macrocycle around the NH centre of a secondary amine. In this way, a simple Lewis base was converted into a sterically encumbered one, which when combined with a bulky Lewis acid, B(C_6_F_5_)_3_, created a Frustrated Lewis Pair (FLP) capable of inducing the heterolytic activation of hydrogen gas.^13^ More recently, Leigh has shown that switching of the selectivity of a rotaxane catalyst can be achieved by controlling the position of the macrocyclic wheel on the axle.^14a,b^ Moreover, Berna found that a rotaxane structure can promote the regioselectivity of an intramolecular ring closure reaction.^14*c*^

**Fig. 1:**
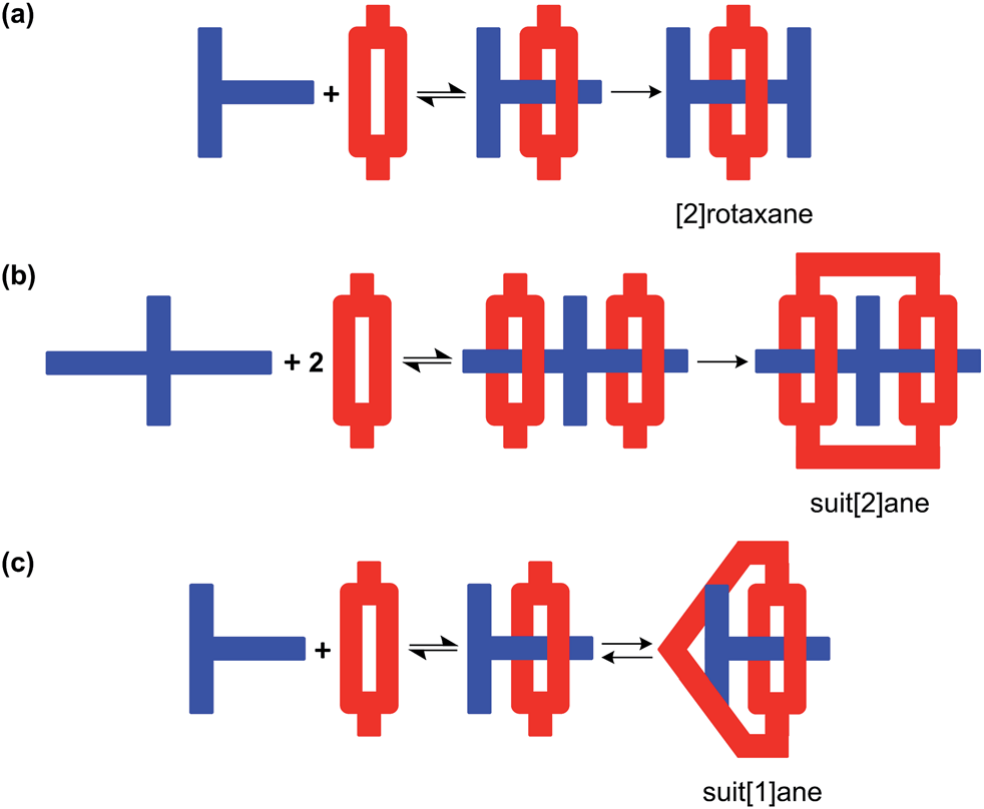
(a) Conversion of an axle to a [2]pseudorotaxane followed by kinetic trapping as a [2]rotaxane, (b) conversion of an axle with two limbs to a [3]pseudorotaxane followed by trapping as a suit[2]ane and (c) conversion of an axle with one limb to a [2]pseudorotaxane followed by trapping as a suit[1]ane – and the reverse. For suit[*n*]anes, the value of *n* represents the number of “limbs” on the axle which are then suited.^15*a*^ This is different from the common designation of *n* as the number of components of an interlocked molecule as in [*n*]rotaxane and [*n*]catenane.

Despite these recent developments, the scope of using MIM formation as a protecting methodology is limited. Herein, we demonstrate that a T-shaped axle can be incorporated into a [2]pseudorotaxane by penetrating a macrocycle and then the macrocycle converted into a cryptand to yield a mechanically interlocked molecule known as a suit[1]ane; Fig. 1.^15^ Furthermore, we show that this “suit” can protect the structure of the axle from external reagents and then subsequently be removed to re-expose the axle, making this 3D protection strategy wholly reversible.

**Scheme 1:**
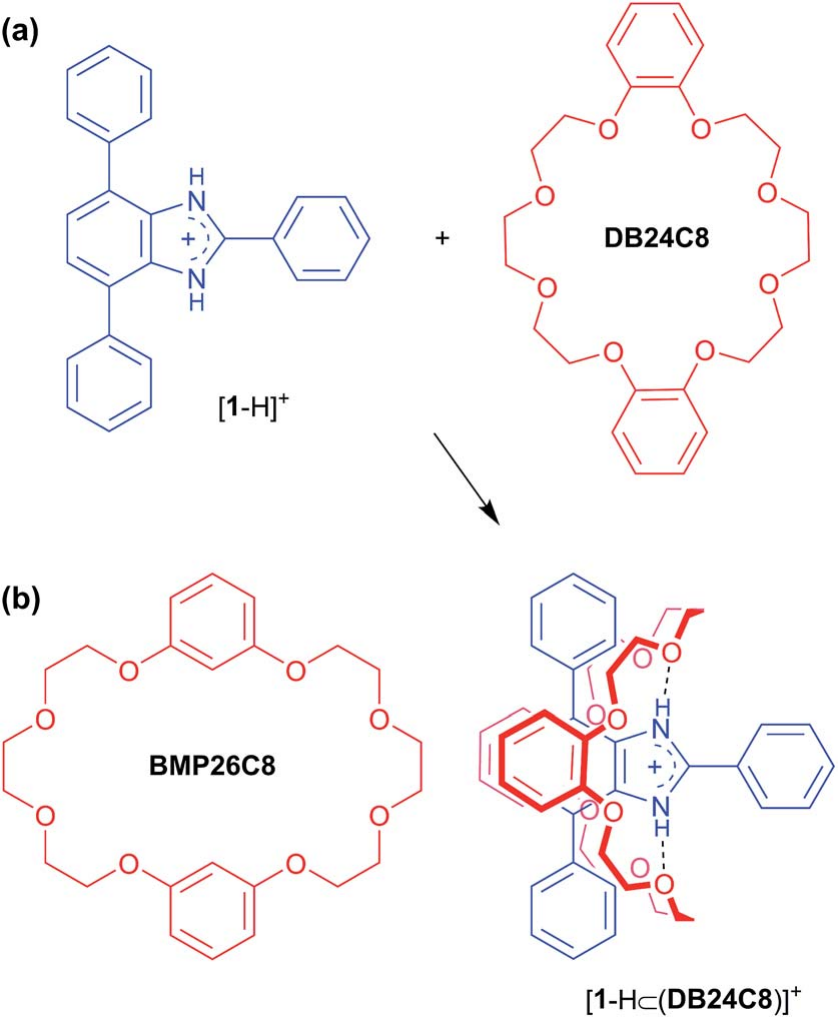
(a) The [2]pseudorotaxane, [**1**-H⊂**DB24C8**]^+^ can be formed *via* supramolecular interactions (ion-dipole, hydrogen-bonding and π-stacking) between the T-shaped benzimidazolium cation, [**1** – H]^+^ and the 24-membered crown ether **DB24C8**.^17,18a^ (b) Of relevance to this work, the crown ether **BMP26C8** can also form a similar [2]pseudorotaxane, [**1**-H⊂**BMP26C8**]^+^ with [**1** – H]^+^.^18*b*^

In addition to secondary ammoniums and pyridiniums, it is now well established that imidazolium and benzimidazolium cations also complex well with crown ether hosts.^16^ In this regard, we developed a T-shaped benzimidazolium cation [**1** – H]^+^ which can act as an axle for the formation of [2]pseudorotaxanes with a wide variety of crown ethers including **DB24C8** and **BMP26C8**.^17^ In the X-ray structure of [2]pseudorotaxane [**1**-H⊂**DB24C8**]^+^, the crown ether was found to be clamped around the T-shaped axle using ion-dipole, hydrogen-bonding and π-stacking interactions; Scheme 1. This interpenetrated structure could then be trapped by stoppering the end of the axle to form a [2]rotaxane; Fig. 1a.^18^

We propose that this clamped structure could be further employed to construct a suit[1]ane by bridging the two aromatic rings of the crown ether with a third linking chain; Fig. 1c.^19^ To this end, we report herein: (1) the synthesis and properties of suit[1]anes based on the crown ether/benzimidazolium recognition motif, (2) evidence that this type of encapsulation can be used to protect the axle unit from the interaction with external reagents; in this case, strong base and (3) that the formation of the protective three-dimensional cage can be made reversible using ring opening metathesis – *i.e.* the suit can be removed when the protection is no longer required.

## Results and discussion

**Scheme 2:**
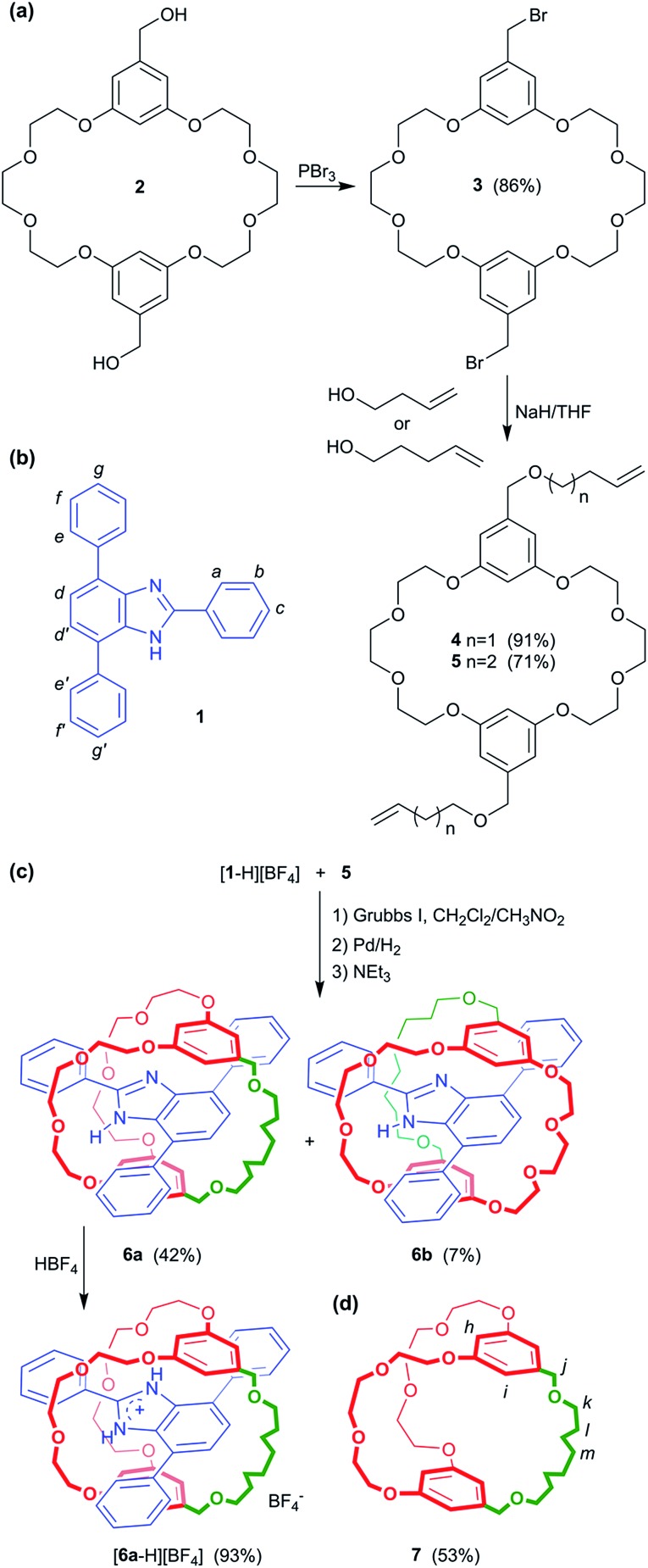
(a) Preparation of macrocycles **4** and **5** appended with olefinic groups. (b) T-shaped benzimidazole **1** with labelling scheme for ^1^H NMR spectra. (c) Template-directed synthesis of isomeric suit[1]anes **6a** and **6b** from the [2]pseudorotaxane [**1**-H⊂**5**]^+^ under ring-closing metathesis conditions and subsequent protonation of **6a** to give [**6a** – H][BF_4_]. (d) Free macrobicycle **7** can be prepared under the same conditions in the absence of axle template [**1** – H]^+^; labelling scheme for ^1^H NMR spectra is shown.

**Fig. 2:**
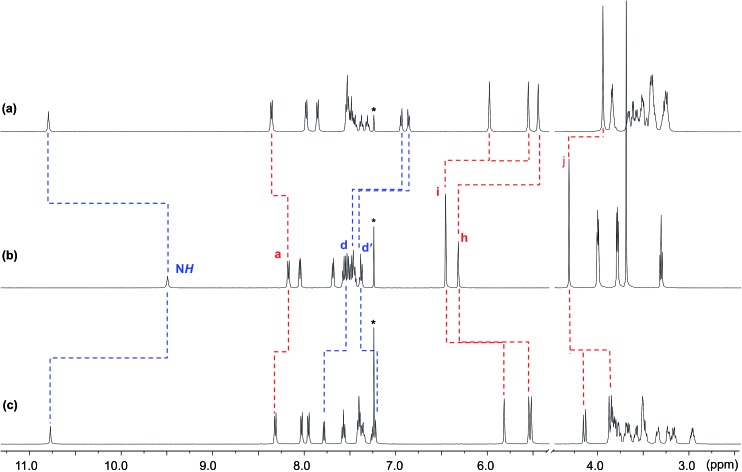
Comparison of the partial ^1^H NMR spectra (500 MHz, CDCl_3_) of (a) suit[1]ane **6a**, (b) an equimolar solution of **1** and **7** and (c) suit[1]ane **6b** (* = CHCl_3_). Labelling scheme is shown in Scheme 2 for **1** and **7**.

The crown ether **BMP26C8** – rather than **DB24C8** – was chosen as the parent macrocycle for fabrication of the suit[1]ane. Although, the initial association constant for [2]pseudorotaxane formation was lower for **BMP26C8** (210 *vs.* 1970 M^–1^ for **DB24C8** in CD_3_CN),^18*b*^ the *meta* substitution pattern greatly simplified the incorporation of the third chain from a synthetic point-of-view. The target suit[1]ane **6a** was prepared as outlined in Scheme 2. The T-shaped benzimidazolium axle [**1** – H][BF_4_] was readily prepared using a previously reported method^17^ and the appended crown ethers **4** and **5** were constructed *via* multiple-step syntheses. The bis(hydroxymethyl) substituted crown ether **2** was prepared according to a literature method^20^ and brominated to afford the bis(bromomethyl) crown ether **3** which was alkylated to afford macrocycles **4** and **5** in yields of 91% and 71%, respectively. This was followed by a Grubbs' I catalysed ring closing metathesis (RCM) reaction^21^ to construct the suit[1]ane. No evidence of suitane formation was observed when macrocycle **4** was used indicating the appendages were not long enough to fully encapsulated the benzimidazolium axle, however, macrocycle **5** with longer appendages yielded the desired product. Subsequent hydrogenation with H_2_(g) and neutralization with triethylamine afforded the target suit[1]ane **6a** in 42% yield; isomeric suitane **6b** (7%) was isolated as a by-product, along with a small amount of free cryptand **7** (<10% by NMR). A comparison of the ^1^H NMR spectra of suit[1]anes **6a**, **6b** and free components **1** and **7** is shown in Fig. 2. The spectrum of **6a** (Fig. 2a) is very different from an equimolar mixture of the non-interlocked components **1** and **7** (Fig. 2b). The downfield chemical shifts observed for the NH and a protons of the benzimidazole axle at 10.77 and 8.31 ppm respectively are attributed to hydrogen-bonding between these protons and crown ether O-atoms. The large upfield chemical shifts of the aromatic protons h, i, d and d′ indicate the crown ether is clamped around the axle in a fashion similar to that observed previously for [2]pseudorotaxanes and [2]rotaxanes involving a benzimidazolium axle.^18*b*^ In the suit[1]ane **6a**, the newly formed covalent link makes this clamped structure permanent even after the axle has been neutralized. Interestingly, due to the unsymmetrical nature of neutral **1**, the aromatic proton i is split into two different signals at 5.95 and 5.58 ppm in the interlocked structure as compared to the 1 : 1 mixture of **1** and **7**. The ^1^H NMR spectrum of **6b** is shown in Fig. 1c. Although a similar chemical shift change is observed for the NH proton, the other protons show very different signals. For example, compared to the singlet at 3.89 ppm in **6a**, the methylene proton signal j in **6b** is split into two coupled doublets at 4.19 ppm and 3.90 ppm, clearly indicating different chemical environments for the two linkages.

The existence of two isomeric suit[1]anes, **6a** and **6b**, results from macrocycle **5** threading over, not only the phenyl ring substituted at the 2-position of the benzimidazolium cation as predicted, but also the phenyl ring substituted at the 4- (and 7-) position – albeit to a much lower extent.

Surprisingly, only one set of signals was observed for **6b**. Commonly, two structures are observed for these types of neutral axles due to slow tautomerization on the NMR time scale,^22^ but in this case, after formation of the interlocked suit[1]ane, the NH proton is immobilized due to hydrogen-bonding to the cryptand.

The isomeric structures **6a** and **6b** were further identified by 2D NOESY experiments which allowed determination of the different spatial arrangements of the axle inside the cryptand; Fig. S1.† Cross peaks between alkane proton m of the cryptand and aromatic protons d and d′ of the axle were observed for **6a**, while for **6b**, proton m was found to be spatially closer to aromatic protons e and f. This is consistent with the structural assignments made from the 1D ^1^H NMR spectra shown in Fig. 2.

**Fig. 3:**
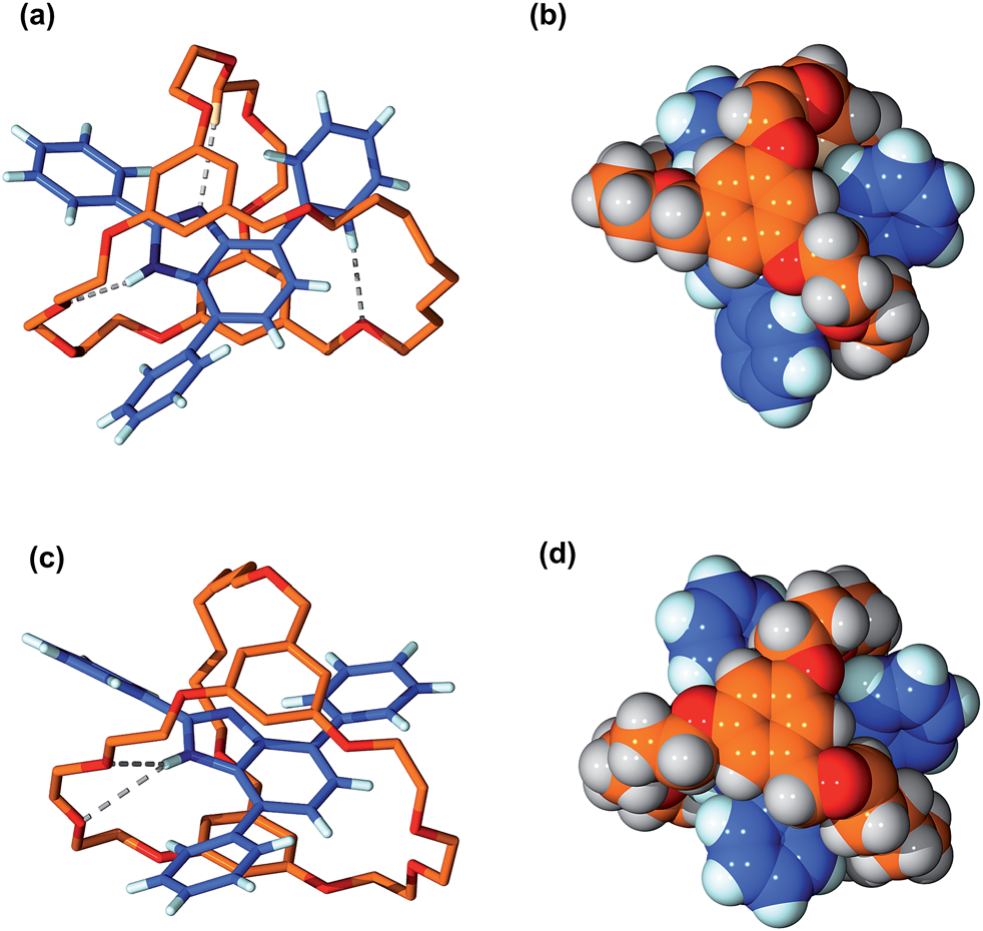
Single-crystal X-ray structure of suit[1]ane **6a**, (a) wire-stick representation and (b) space-filling model. Single-crystal X-ray structure of suit[1]ane **6b**, (c) wire-stick representation and (d) space-filling model. Colour code: O = red, N = dark blue, C of axle = blue, C of cryptand = orange, H = white. For wire-stick models, hydrogens not involved in H-bonding are omitted for clarity.

The structures of suit[1]anes **6a** and **6b** were further confirmed by single-crystal X-ray diffraction;† representations of the solid state structures are shown in Fig. 3. For the structure of **6a**, three hydrogen bonds were found to be the main residual interactions between the two independent components. The benzimidazole NH is clearly hydrogen bonded to an O-atom of the cryptand with a N···O distance of 2.99 Å and an N–H···O angle of 163°. In addition one of the polyether methylene protons forms a C–H···N interaction with the basic N-atom of the benzimidazole moiety. The aromatic group of the benzimidazole and one of the aromatic rings of the cryptand were found to be approximately parallel with a distance of *ca.* 3.53 Å indicative of π-stacking. This π-stacking is also observed in the structure of **6b**, but only two hydrogen bonds between the benzimidazole NH proton and cryptand O-atoms were observed. This is presumably due to the different orientation of the T-shaped axle with respect to the polyether chains of the cryptand.

It is clear from the space filling models, that for both structures, the NH protons are completely buried inside the cryptand. This is an important observation and provides preliminary evidence that the benzimidazole axles of the suit[1]anes may have different reactivity when compared to the free benzimidazole **1**.

In order to further prove the suit[1]anes are truly mechanically interlocked molecules, both **6a** and **6b** were dissolved in DMSO-*d*_6_ and heated to 100 °C for 24 h; conditions that are known to result in unthreading *via* slippage for some purported [2]rotaxanes.^23^ No evidence for disassociation of the assemblies was observed in the ^1^H NMR spectra under these relatively harsh conditions when compared to an equimolar solution of **1** and **7**; Fig. S2.†

Benzimidazole has potential for the fabrication of fluorescent polymers due to its versatile electronic structure;^24^ the anionic form is especially useful and can be accessed by deprotonation with strong base.^25^ It was therefore of interest to compare the optical properties of the free T-shaped benzimidazole **1** with that of the same molecule encapsulated inside the suit[1]ane, **6a**.

**Fig. 4:**
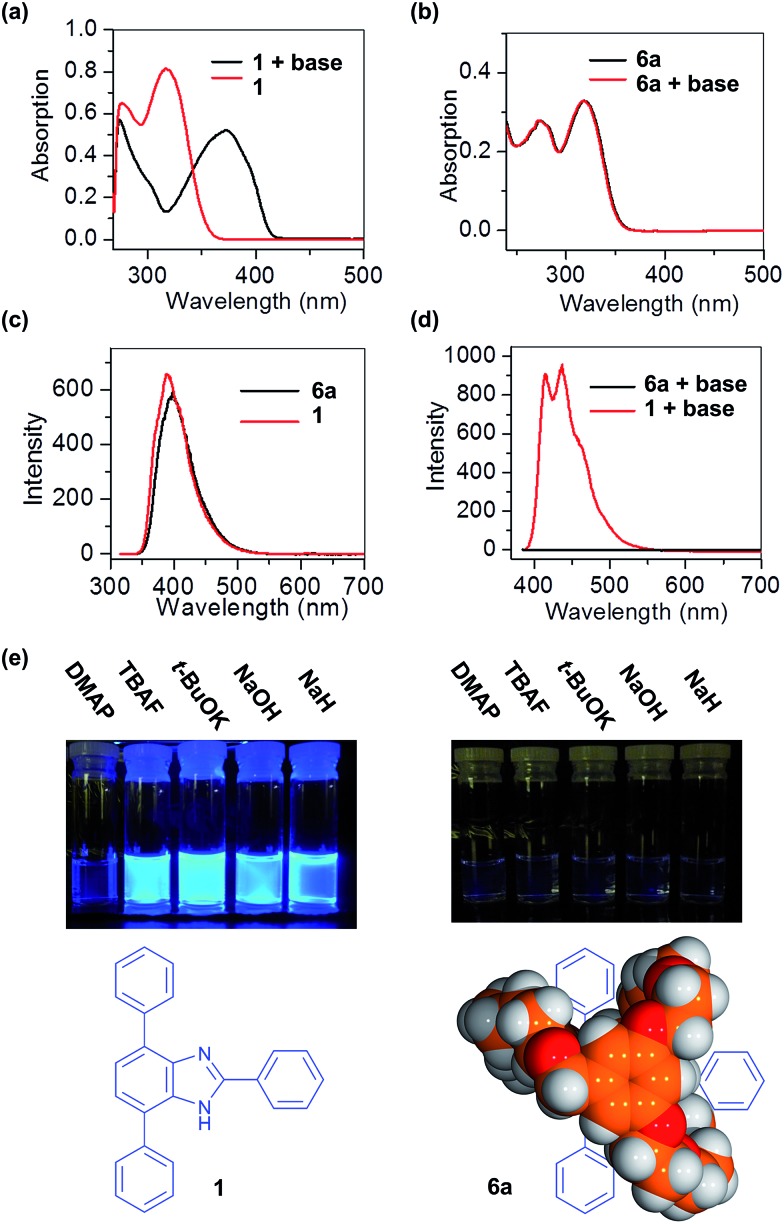
(a) UV/vis spectra of neutral and anionic **1**, (b) UV/vis spectra of **6a** and **6a** after treatment with 2.0 equiv. of potassium *tert*-butoxide, (c) fluorescence spectra of **1** and **6a** when excited at 320 nm, (d) fluorescence spectra of **1** and **6a** after treatment with 2.0 equiv. of potassium *tert*-butoxide when excited at 381 nm, (e) deprotonation of **1** and **6a** with various strong bases and then irradiated at 365 nm using a UV lamp (DMAP = 4-dimethylaminopyridine, TBAF = tetrabutylammonium fluoride, solvent = THF).

When **1** was treated with excess potassium *tert*-butoxide in THF, the maximum absorption band, *λ*_max_, shifted from 322 to 381 nm; Fig. 4a. Neutral **1** also has a fluorescence emission band at 389 nm when excited at 320 nm; Fig. 3c. After deprotonation, the anionic **1** exhibits strong emission bands at 424 and 449 nm when excited at 381 nm; Fig. 3d. This large wavelength shift can be attributed to the release of the steric repulsion between the N–H and the adjacent C–H from the substituted phenyl group.^26^ In addition, complete disappearance of the NH signal from the ^1^H NMR spectrum provides further evidence for deprotonation of the benzimidazole unit; Fig. S3.†

The suitane **6a** has a maximum absorption band *λ*_max_ at 328 nm in THF which is very similar to that of the free T-shaped benzimidazole **1**; Fig. 3b. It also has a fluorescence emission band at 395 nm when excited at 320 nm; Fig. 3c. However, after adding excess potassium *tert*-butoxide to the solution of **6a**, no significant change in the UV/vis or fluorescence spectra were observed. This clearly indicates that deprotonation of the benzimidazole unit inside **6a** does not occur under these strongly basic conditions. A series of other moderate and strong bases (DMAP, TBAF, NaOH and NaH) were also screened, but again, no deprotonation was observed.^27^ The best explanation for these result is that the encapsulation of the benzimidazole by the cryptand can sterically protect the NH group and prevent deprotonation.^28^ This lack of reactivity was also observed by monitoring the reactions by ^1^H NMR spectroscopy; no changes were observed for the ^1^H NMR spectra of a sample of **6a** when treated with excess base; Fig. S4.†

**Fig. 5:**
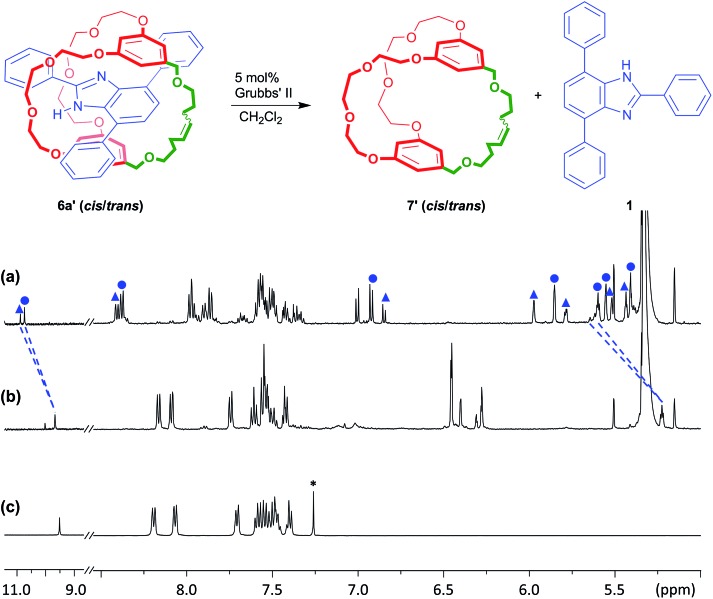
Removing the ‘suit’ by Ru(ii) catalysed ring opening metathesis (ROM). Comparison of the partial ^1^H NMR spectra of: (a) 2.0 mM solution of suit[1]ane **6a′** in CD_2_Cl_2_, (b) solution from (a) with 5 mol% Grubbs II cat. added and (c) free thread **1** in CDCl_3_ (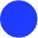 = *trans*, 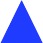 = *cis*, 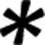 = CHCl_3_).

Although the protection offered by the cryptand to the benzimidazole group inside suit[1]ane **6a** is interesting, traditional protecting groups used in organic synthesis can be installed and then readily removed after the synthetic transformation of interest has been achieved. As such, we were encouraged to find a way to make the “suiting up” of axle **1** demonstrated herein reversible as well. To this end, it occurred to us that for the neutral suit[1]ane, loss of the templating charge should result in a repulsion between the two neutral components – axle and cryptand – thus making the resulting [2]pseudorotaxane thermodynamically unfavourable should one of the chains of the cryptand be broken; Fig. 5. We thus used RCM to prepare the unsaturated version of the suitane **6a**, **6a′** (*cis*/*trans* = 1 : 2.1) and used this as the protected species, with the idea of being able to remove the “suit” *via* ring opening metathesis (ROM) chemistry.^29^

A sample of suitane **6a′** was reacted with 5 mol% Grubbs' II catalyst and after stirring at 25 °C for 10 min, the ^1^H NMR spectrum was recorded. As shown in Fig. 5b, both the *cis*/*trans* isomers of **6a′** have disappeared and merged to one set of signals which are very similar in chemical shift to that observed for the free T-shaped benzimidazole axle **1**. It was also noted that aromatic protons h, i, and j show similar chemical shifts to those observed for the reduced cryptand **7**. This infers that the ROM catalysis produces mostly cryptand rather than oligomers or polymers; this may be due to the relatively low concentration (2.0 mM) used for the reaction.

## Conclusions

Utilising the benzimidazolium/crown ether recognition motif, we have templated the synthesis of a unique MIM – a suit[1]ane – comprised of a T-shaped axle encapsulated inside a cryptand. It was further demonstrated that surrounding the benzimidazole axle with the cryptand “suit” protects the axle from the effects of a reagent such as a strong base. Moreover, this novel three-dimensional protecting group could be easily removed making this a reversible process facilitated by catalytic RCM and ROM. This concept of reversible mechanical protection may have potential applications in synthetic organic chemistry, and the synthesis of complicated systems such as molecular machines in which the rigid interlocked nature of the suit[1]ane might be coupled to the dynamics of rotaxanes and catenanes.
