# Supplementary material for: Reversible mechanical protection: building a 3D “suit” around a T-shaped benzimidazole axle
Source: Chem Sci. 2017 Mar 28;8(5):3898–904. doi: 10.1039/c7sc00790f (PMC5465563; doi:10.1039/c7sc00790f)
Supplement: Supplementary file 1 [file SC-008-C7SC00790F-s001.pdf]

Electronic Supplementary Material (ESI) for Chemical Science.
This journal is © The Royal Society of Chemistry 2017

Supporting information for:

Reversible mechanical protection: building a 3D "suit" around a T-shaped benzimidazole axle

Kelong Zhu[a], Giorgio Baggi[b], V. Nicholas Vukotic[b], and Stephen J. Loeb[b]**

[a] School of Chemistry, Sun Yat-Sen University, Guangzhou, P. R. China 510275.

[b] Department of Chemistry and Biochemistry, University of Windsor, Windsor, Ontario, Canada N9B 3P4

**Table of Content**	**Page**
General comments	S2
Synthesis of **3**	S2
Synthesis of **4**	S3
Synthesis of **5**	S3
Synthesis of **7**	S4
Synthesis of **6a** and **6b**	S4
Synthesis of [**6a**-H][BF_4]	S5
**Figure S1**. ^{1}H- ^{1}H NOESY spectra of **6a** and **6b**	S6
**Figure S2**. ^{1}H NMR of suitane in DMSO-d_6	S7
**Figure S3**. ^{1}H NMR spectra of **1** with tert-BuOK	S8
**Figure S4**. ^{1}H NMR spectra of **6a** with tert-BuOK	S8
X-ray Crystallography	S9
**Table S1**. Summary of Single Crystal X-ray Study for **6a** and **6b**	S10
NMR Spectra of Compounds	S11
References	S17

General comments:

All chemicals were purchased from Aldrich Chemicals and used without further purification. Solvents were dried using an Innovative Technologies Solvent Purification System. Thin layer chromatography (TLC) was performed using Teledyne Silica gel 60 F254 plates and viewed under UV light. Column chromatography was performed using Silicycle Ultra-Pure Silica Gel (230 – 400 mesh). The solvents were dried and distilled prior to use. NMR spectra were recorded on Bruker Advance 500 locked to the deuterated solvent operating at 500 MHz. Mass spectra were performed on a Micromass LCT electrospray ionization TOF spectrometer. Solutions with concentrations of 0.001 molar were prepared in methanol and injected for analysis at a rate of 5 μL/min using a syringe pump. UV/Visible spectra were obtained on a Varian Cary 50 UV/vis Spectrophotometers. Fluorescence spectra were obtained on a Varian Cary Eclipse fluorescence spectrophotometer. All single crystal X-ray data were collected on a Bruker APEX diffractometer with a CCD detector operated at 50 kV and 30 mA with MoKα radiation. Crystals were frozen in paratone oil inside a cryoloop and reflection data were integrated from frame data obtained from hemisphere scans. Compounds 3,6-bis(phenyl)-1,2-diaminobenzene,[S1] 2,4,7-triphenylbenzimidazole, **1**,[S2] 2,4,7-triphenylbenzimidazolium tetrafluoroborate [**1**-H][BF_4],[S2] and bis(5-hydroxymethyl-*m*-phenylene)[26]crown-8, **2**,[S3] were synthesized using reported literature methods.

Synthesis of Bis(5-bromomethyl-m-phenylene)[26]crown-8, (3)

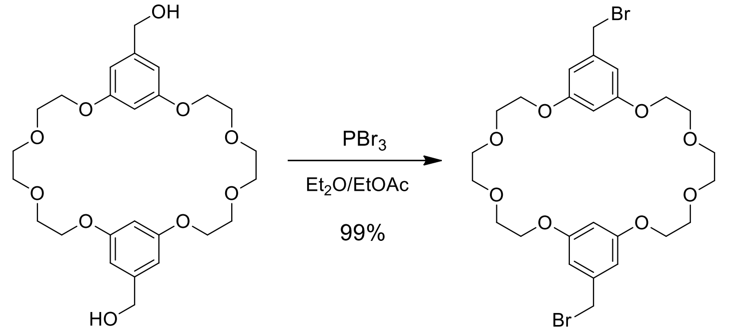

Phosphorus tribromide (1.0 M in dichloromethane, 29.5 mL, 29.5 mmol) was slowly added to a stirring mixture of bis(5-hydroxymethyl-*m*-phenylene)[26]crown-8 (**2**) (2.5 g, 4.92mmol), diethyl ether (250 mL), and ethyl acetate (30 mL). After stirring for 24 h at room temperature, to the mixture was added CH_2Cl_2 (50 mL) and then the solution carefully poured on to ice-water (100 g). The organic phase was separated, washed with saturated sodium bicarbonate solution (x1) and brine (x1). After drying over anhydrous sodium sulphate, the solvent was removed on a rotary evaporator. The oily residue solidified after the addition of ethyl acetate (10 mL). The solid was collected by filtration, rinsed with ether and air dried. Yield: 3.10 g, 99%. Mp 129–131 °C. The product was utilised in the next step without further purification. ^{1}H NMR (500 MHz, $CDCl_3$, 298 K) δ= 6.52 (s, 4H), 6.41 (s, 2H), 4.37 (s, 4H), 4.06 (m, 8H), 3.83 (m, 8H), 3.72 (s, 8H). ^{13}C NMR (125 MHz, $CDCl_3$, 298 K) δ= 160.0, 139.6, 107.8, 101.9, 71.0, 69.6, 67.5. HRMS (ESI): 635.0678 calcd for $C_{26}H_{35}Br_2O_8$ $[M+H]^+$, found: 635.0669.

Synthesis of (4)

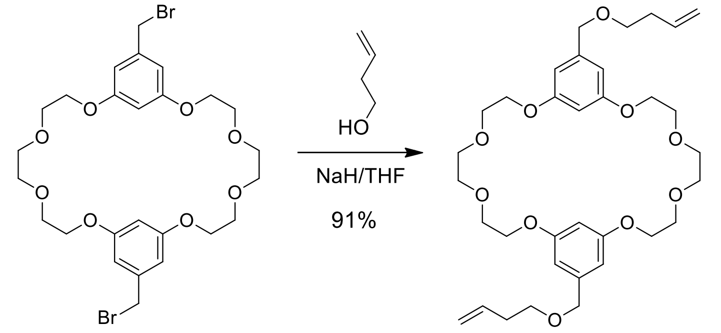

Sodium hydride (60% in mineral oil, 19 mg, 0.473 mmol) was added to a Schlenk flask under nitrogen atmosphere. After addition of a small amount of THF (5 mL), a solution of 3-buten-1-ol (34 mg, 0.473 mmol) in THF (2 mL) was added dropwise to the mixture. After cessation of bubbling, the mixture was allowed to stir for 1 h at room temperature. A solution of **3** (100 mg, 0.158 mmol) in THF (15 mL) was then introduced to the mixture in portions by syringe. The mixture was further stirred at room temperature for 2 days and quenched by carefully addition of water dropwise. After ethyl acetate (15 mL) and brine (20 mL) were added, the organic layer was separated and dried over anhydrous magnesium sulphate. Removal of the solvent gave a viscous oil which was purified by column chromatography (SiO_2, ethyl acetate/hexane= 1:1). Colorless oil. Yield: 89 mg, 91%. ^{1}H NMR (500 MHz, $CDCl_3$, 298 K) δ = 6.49 (s, 4H), 6.42 (s, 2H), 5.82 (m, 2H), 5.08 (m, 4H), 4.42 (s, 4H), 4.08 (m, 8H), 3.85 (m, 8H), 3.73 (s, 8H), 3.48 (t, 4H, *J* = 4.8 Hz), 2.36 (q, 4H, *J* = 4.8 Hz). ^{13}C NMR (125 MHz, $CDCl_3$, 298 K) δ = 160.1, 141.0, 138.4, 114.7, 106.2, 100.9, 72.8, 71.0, 69.7, 69.6, 67.4, 31.3. HRMS (ESI): 617.3326 calcd for $C_{34}H_{49}O_{10}$ $[M+H]^+$, found: 617.3329.

Synthesis of (5)

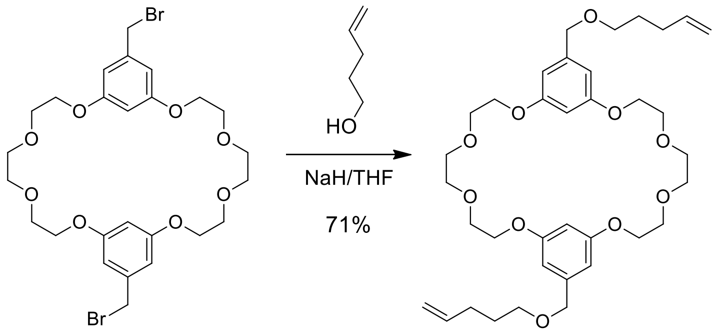

This compound was prepared according to the procedure used for compound **4**. Sodium hydride (60% in mineral oil, 640 mg, 16 mmol) was added to a Schlenk flask (250 mL) under an N_2 atmosphere. After addition of a small amount of THF (10 mL), a solution of 4-penten-1-ol (1.38 g, 16 mmol) in THF (20 mL) was added dropwise to the mixture. After cessation of bubbling, the mixture was allowed to stir for 1 h at room temperature. A solution of **3** (2.54 g, 0.158 mmol) in THF (60 mL) was then introduced to the mixture in portions *via* syringe. The mixture was further stirred at room temperature for 1 day and

quenched by carefully adding water dropwise. After ethyl acetate (150 mL) and brine (150 mL) were added, the organic layer was separated and dried over anhydrous magnesium sulphate. Removal of the solvent gave a viscous oil which was purified by column chromatography (SiO_2, ethyl acetate/hexane = 1:1). Yield: 1.84 g, 71%. ^{1}H NMR (500 MHz, $CDCl_3$, 298 K) δ = 6.49 (s, 4H), 6.42 (s, 2H), 5.82 (m, 2H), 5.08 (m, 4H), 4.40 (s, 4H), 4.08 (m, 8H), 3.84 (m, 8H), 3.73 (s, 8H), 3.44 (t, 4H, *J* = 4.8 Hz), 2.13 (q, 4H, *J* = 4.8 Hz), 1.69 (m, 4H). ^{13}C NMR (125 MHz, $CDCl_3$, 298 K) δ = 160.0, 141.0, 138.3, 114.7, 106.1, 100.9, 72.8, 71.0, 69.7, 69.6, 67.4, 30.4, 29.0. HRMS (ESI): 645.3639 calcd for $C_{36}H_{53}O_{10}$ $[M+H]^+$, found: 645.3645.

Synthesis of (7)

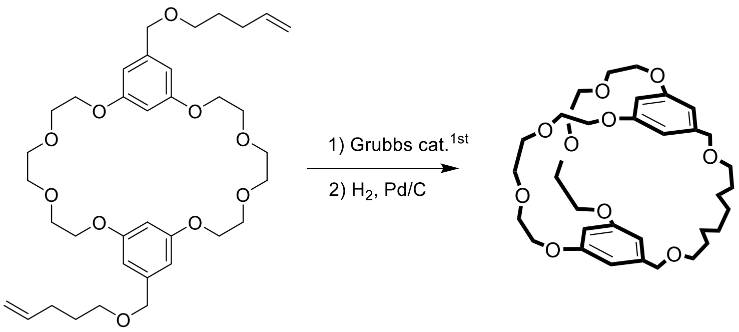

A mixture of **5** (100 mg, 0.156 mmol) and 1st generation Grubbs' catalyst (13 mg, 0.016 mmol) in dichloromethane (50 mL) was refluxed at 45 ºC overnight. After the solvent was evaporated, the dark oily residue was dissolved in methanol (20 mL) and added to a Schlenk flask which was equipped with 10% Pd/C (33 mg, 0.031 mmol) under an N_2 atmosphere. The flask was slightly degassed and flushed and H_2 introduced *via* a balloon. The mixture was stirred for 3 h under ambient conditions. Solvent was then evaporated on a rotary evaporator to give a dark residue which was separated using column chromatography (SiO_2, CH_2Cl_2/ethyl acetate = 1:2). Yield 79 mg, 53%. ^{1}H NMR (500 MHz, $CDCl_3$, 298 K) δ = 6.48 (s, 4H), 6.34 (s, 2H), 4.33 (s, 4H), 4.01 (m, 8H), 3.81 (m, 8H), 3.71 (s, 8H), 3.33 (t, 4H, *J* = 4.8 Hz), 1.47 (m, 4H), 1.21 (m, 4H), 1.11 (m, 4H). HRMS (ESI): *m/z* 619.3482 calcd for $C_{34}H_{51}O_{10}$ $[M + H]^+$; found: 619.3476.

Synthesis of (6a) and (6b)

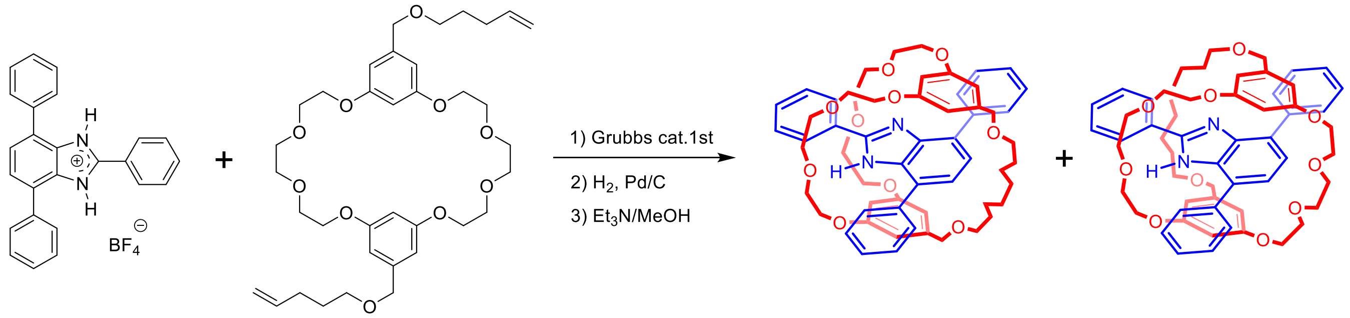

A mixture of [**1**-H][BF_4] (217 mg, 0.5 mmol) and **5** (387 mg, 0.6 mmol) in dichloromethane/nitromethane (9:1, 15 mL) was stirred at room temperature until a clear solution was formed. After adding 1st generation Grubbs' catalyst (41 mg, 0.05 mmol), the mixture was heated at 43 ºC overnight. An

additional portion of catalyst (20 mg, 0.025 mmol) was added and heated at 43 ºC for another day. After the solvent was evaporated, the dark oily residue was dissolved in methanol (20 mL) and added to a Schlenk flask which was equipped with 10% Pd/C (106 mg, 0.1 mmol) under N_2 atmosphere. The flask was slightly degassed and flushed and H_2 introduced *via* a balloon. The mixture was stirred for 3 h under ambient conditions. After the mixture was filtered, triethylamine (0.5 mL) was added and then the mixture evaporated on a rotary evaporator to give the dark residue which was separated by column chromatography (SiO_2, CH_2Cl_2/ethyl acetate/triethylamine = 200:100:1 for **6a**; CH_2Cl_2/ethyl acetate = 9:1 for **6b**). **6a**: white solid, yield 203 mg, 42%, Mp: 176–179 °C. ^1H NMR (500 MHz, $CDCl_3$, 298 K) δ = 10.82 (s, 1H), 8.38 (d, 2H, J = 8.0 Hz), 8.00 (d, 2H, J = 8.0 Hz), 7.88 (d, 2H, J = 8.0 Hz), 7.33–7.55 (m, 7H), 7.40 (t, 1H, J = 8.0 Hz), 7.36 (t, 1H, J = 8.0 Hz), 6.95 (d, 1H, J = 7.5 Hz), 6.89 (d, 1H, J = 7.5 Hz), 6.00 (s, 1H), 5.57 (s, 1H), 5.46 (s, 1H), 3.96 (s, 4H), 3.86 (s, 4H), 3.69–3.26 (m, 24H), 1.45–1.64 (m, 12H). ^{13}C NMR (125 MHz, $CDCl_3$, 298 K) δ = 158.7, 157.9, 150.5, 141.1, 140.3, 140.3, 139.9, 130.8, 130.0, 129.1, 129.0, 128.5, 128.4, 128.0, 126.4, 125.8, 122.8, 122.7, 106.6, 103.8, 97.8, 72.4, 71.1, 69.9, 69.8, 69.6, 68.9, 65.7, 65.4, 50.9, 29.6, 28.6, 25.7. HRMS (ESI): *m/z* calcd for $C_{59}H_{69}N_2O_{10}$ $[M + H]^+$ 965.4952; found: 965.4958. **6b**: white solid, yield 34 mg, 7%, Mp: 185–187 °C. ^1H NMR (500 MHz, $CDCl_3$, 298 K) δ = 10.80 (s, 1H), 8.33 (d, 2H, J = 8.0 Hz), 8.05 (d, 2H, J = 8.0 Hz), 7.98 (d, 2H, J = 8.0 Hz), 7.81 (d, 1H, J = 8.0 Hz), 7.59 (t, 2H, J = 7.5 Hz), 7.41–7.44 (m, 6H), 7.37 (m, 1H), 5.84 (s, 1H), 5.57 (s, 1H), 5.54 (s, 1H), 4.17 (d, 2H, J = 8 Hz), 3.71–3.89 (m, 9H), 3.75 (m, 4H), 3.63 (m, 2H), 3.51 (m, 6H), 3.40 (m, 2H), 3.21 (m, 4H), 2.95 (m, 2H), 1.25–1.56 (m, 12H). HRMS (ESI): *m/z* calcd for $C_{59}H_{69}N_2O_{10}$ $[M + H]^+$ 965.4952; found: 965.4957.

Synthesis of [6a-H][BF$_4$]

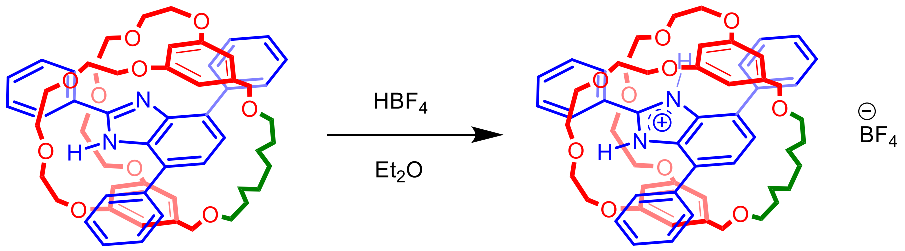

After dissolving **6a** (50 mg, 0.052 mmol) in diethyl ether (20 mL), tetrafluoroboric acid diethyl ether complex (10 μL, 0.074 mmol) was added to the solution using a micro-syringe. The mixture was stirred at room temperature for 5 min. The resulting white precipitate was collected by filtration and air dried. Yield: 51 mg, 93%. Mp: 135–137 °C. ^1H NMR (500 MHz, $CDCl_3$, 298 K) δ = 11.56 (s, 2H), 8.33 (d, 2H, J = 8 Hz), 7.90 (d, 4H, J = 7.5 Hz), 7.87 (m, 3H), 7.64 (t, 4H, J = 7.5 Hz), 7.54 (t, 2H, J = 7.5 Hz), 7.33 (s, 2H), 5.74 (s, 4H), 5.62 (s, 2H), 3.93 (s, 4H), 3.60 (m, 12H), 3.47–3.59 (m, 12H), 3.24 (t, 4H, J = 4.8 Hz), 1.64 (m, 4H), 1.62 (m, 4H), 1.57 (m, 4H). ^{13}C NMR (125 MHz, $CDCl_3$, 298 K) δ = 158.2, 148.5, 140.8, 135.7, 134.9, 130.4, 129.9, 129.4, 129.3, 128.6, 127.9, 126.6, 122.2, 105.5, 97.8, 72.5, 71.7, 70.2, 70.0, 66.4, 29.9, 29.1, 26.9. HRMS (ESI): *m/z* 965.4952 calcd for $C_{59}H_{69}N_2O_{10}$ $[M–BF_4]^+$, found: 965.4958.

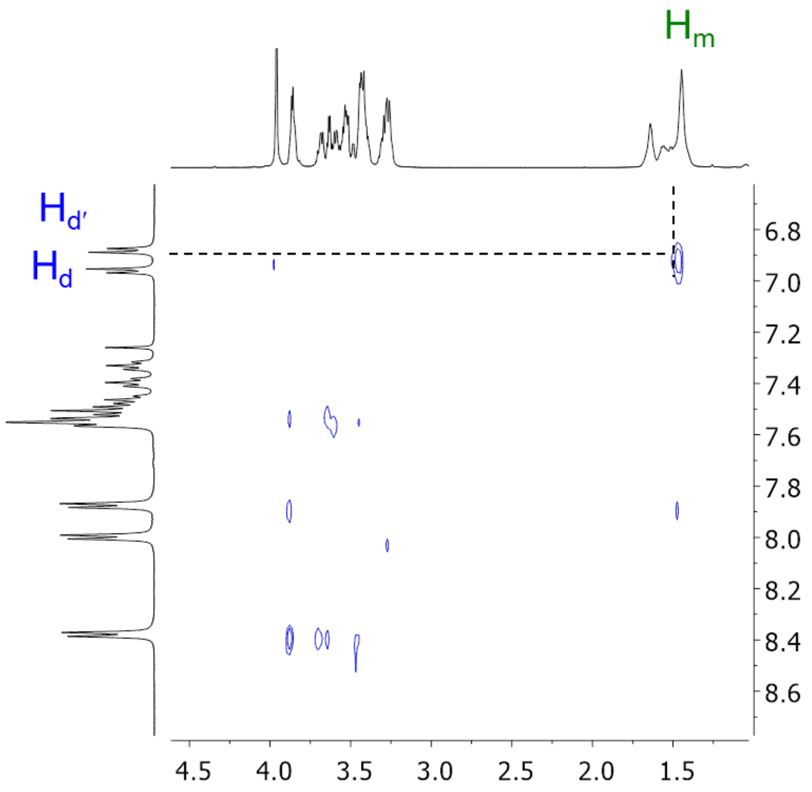

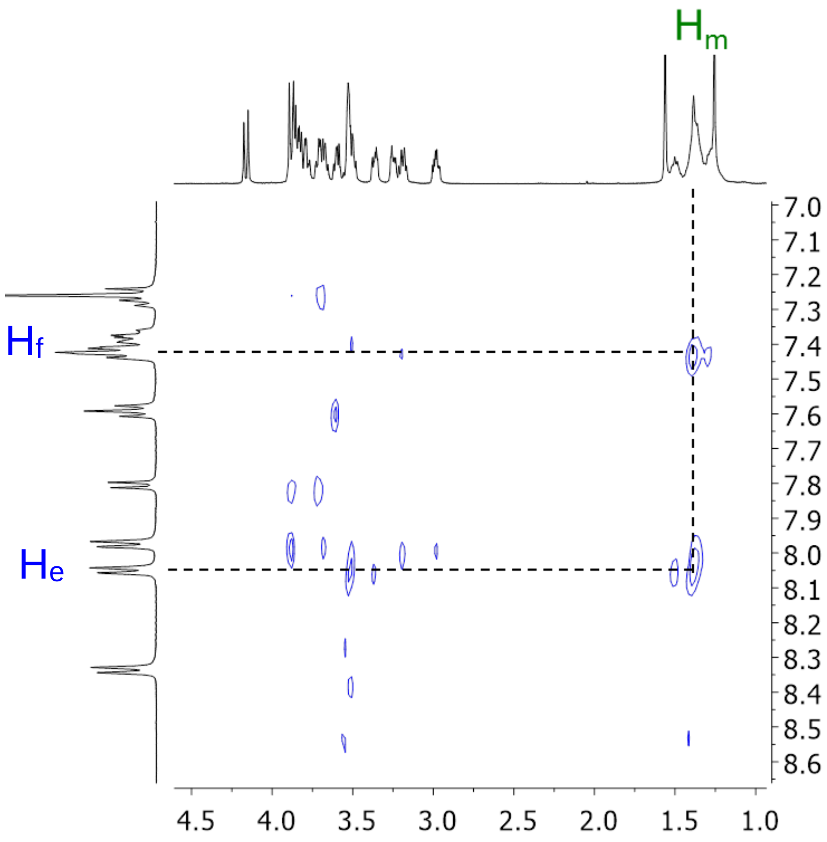

Figure S1. ^{1}H- ^{1}H NOESY spectra of **6a** (top) and **6b** (bottom) in $CDCl_3$.

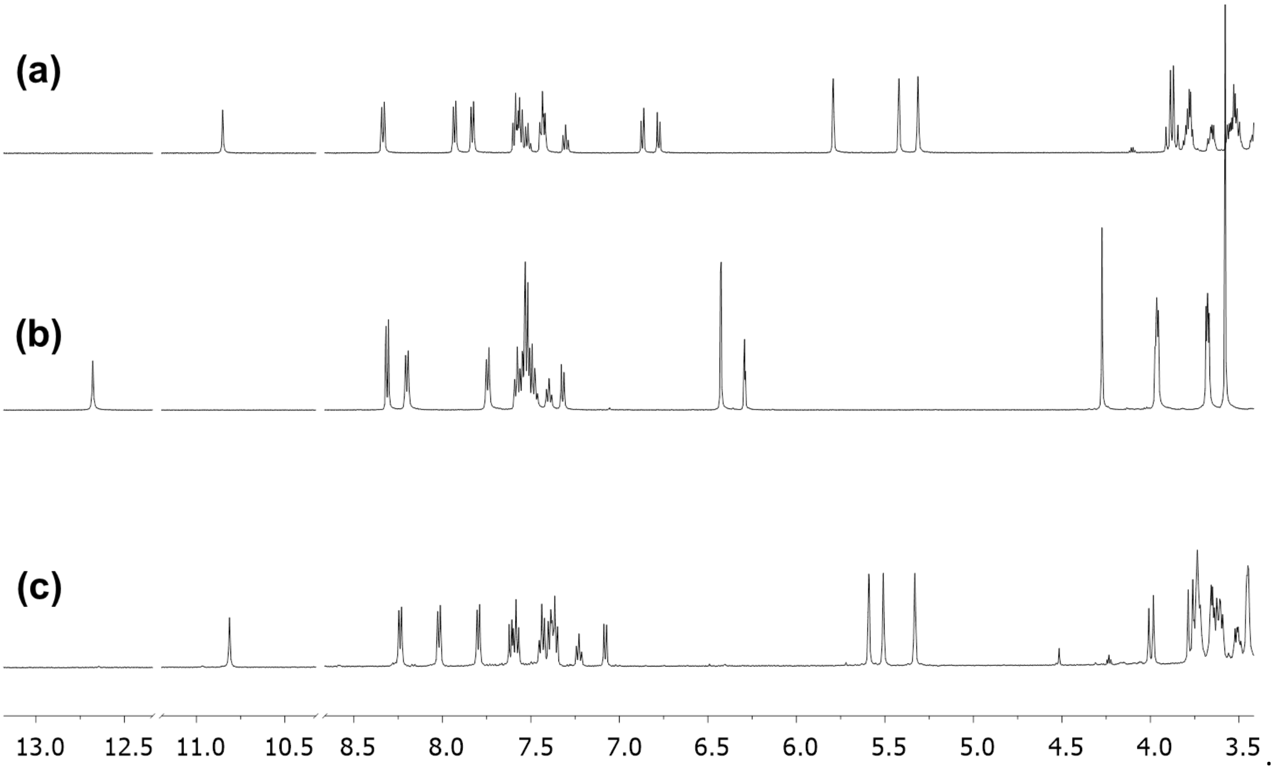

Figure S2. ^{1}H NMR comparison of suit[1]anes and free components in DMSO-d_6. a) **6a**; b) an equimolar mixture of **1** and **7**; and c) **6b**. After heating both suitane isomers samples in DMSO-d_6 at 100 °C for 2 days, no unthreading was observed for either sample of **6a** and **6b**, verifying their permanent interlocked structure.

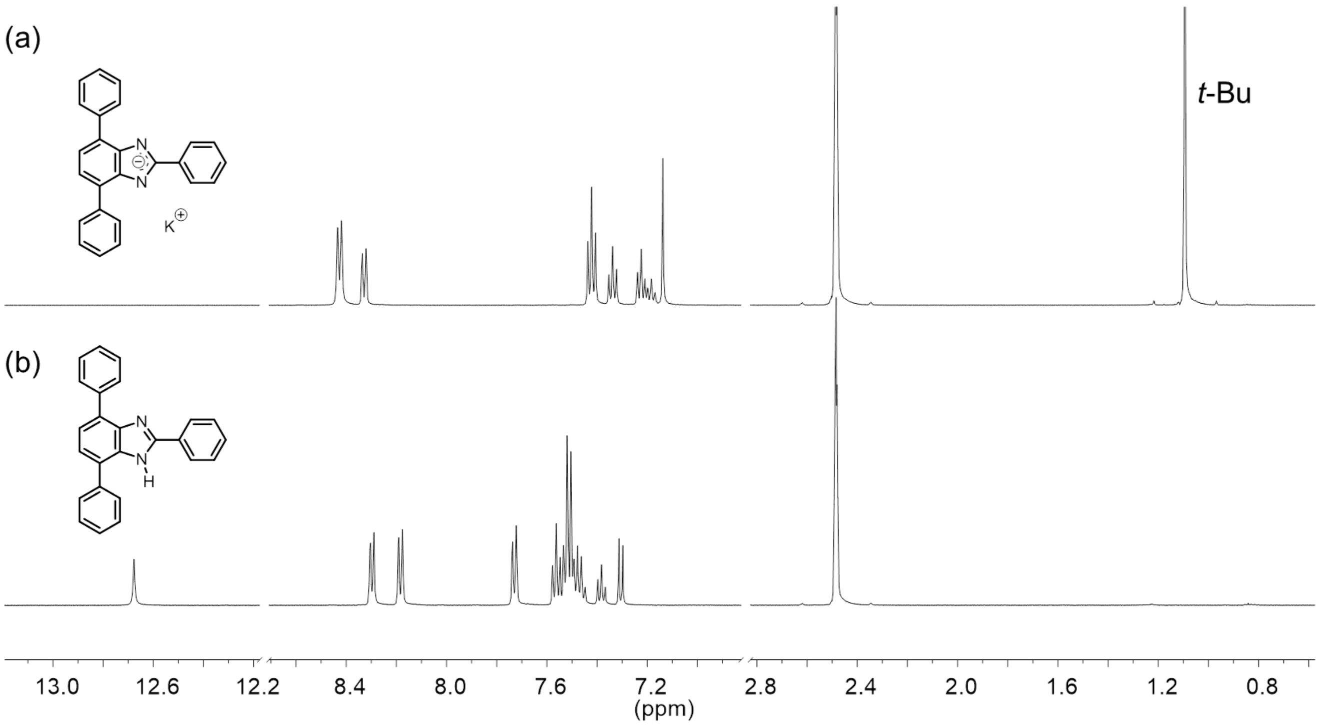

Figure S3. ^{1}H NMR spectra of a) **1** with 1.2 equivalent of *t*-BuOK, and b) **1** in DMSO-d_6.

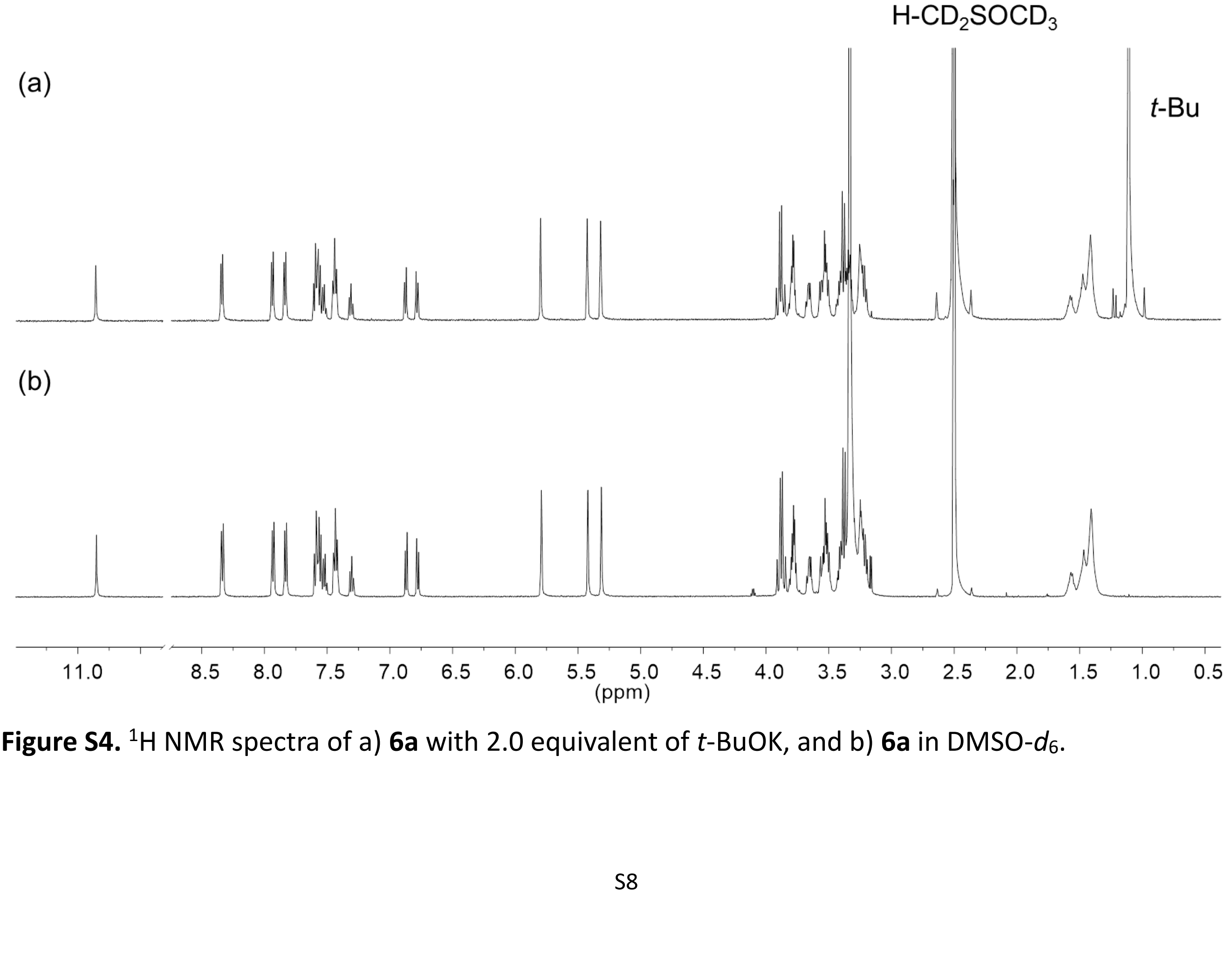

Figure S4. ^{1}H NMR spectra of a) **6a** with 2.0 equivalent of *t*-BuOK, and b) **6a** in DMSO-d_6.

X-ray Crystallography

General: Crystals were frozen in paratone oil inside a cryoloop under a cold stream of N_2. X-ray intensity data were collected at 173(2) K using a Bruker APEX diffractometer equipped with an APEX area detector. The raw area detector data frames were reduced and corrected for absorption effects using the SAINT+ and SADABS programs.[S4] Final unit cell parameters were determined by least-squares refinement taken from the data set. Diffraction data and unit-cell parameters were consistent with the assigned space groups. The structures were solved by direct methods with SHELXT.[S5] Subsequent difference Fourier calculations and full-matrix least-squares refinement against $|F^2|$ were performed with SHELXL-2014[S5] using OLEX2.[S6] All non-hydrogen atoms were refined anisotropically and hydrogen atoms placed in idealized positions and refined using a riding model. See Table S1 for a summary of data collection, solution and refinement details. Complete details of the structures can be obtained from the Cambridge Crystallographic Data Centre at www.ccdc.cam.ac.uk for CCDC accession numbers 1533271 and 1533272.

X-ray structure of Suitane 6a

Single crystals were obtained from slow evaporation of a methanol/H_2O solution of **6a**. Crystals of formula **6a**$(CH_3OH)_2$ were of good quality. Data was collected using MoKα radiation (λ = 0.71073 Å). The asymmetric unit contained one molecule of the suit[1]ane ($C_{59}H_{68}N_2O_{10}$) and two molecules of CH_3OH. The structure was solved in the monoclinic space group $P2_1/n$ (#14). A portion of one of the polyether chains was disordered and restrained with the SAME command and modelled with occupancies of 58:42 using PART and FVAR. See Table S1 for details.

X-ray structure of Suitane 6b

Single crystals were obtained from slow evaporation of an acetonitrile/H_2O solution of **6b.** Crystals of formula **6b**(CH_3CN) were of good quality. Data was collected using MoKα radiation (λ = 0.71073 Å). The asymmetric unit contained one molecule of the suit[1]ane ($C_{59}H_{68}N_2O_{10}$) and one molecule of CH_3CN. The structure was solved in the orthorhombic space group Pbca. A portion of one of the polyether chains and the alkane chain were disordered and restrained with SAME & SIMU commands and modelled with occupancies of 60:40 and 82:18 respectively using PART and FVAR. See Table S1 for details.

Table S1. Single-crystal X-ray data collection, solution and refinement details for **6a** and **6b**.

CCDC No.	1533271	1533272
Compound	**6a**$(CH_3OH)_2$	**6b**(CH_3CN)
Formula	$C_{61}H_{76}N_2O_{12}$	$C_{61}H_{71}N_3O_{10}$
M [gmol^{-1}]	1029.23	1006.20
Crystal system	monoclinic	orthorhombic
Space group	$P2_1/n$ (No. 14)	Pbca (No. 61)
a [Å]	12.9848(10)	17.819(3)
b [Å]	19.3820(15)	17.457(3)
c [Å]	22.2953(17)	34.337(5)
α [°]	90	90
β [°]	99.8421(10)	90
γ [°]	90	90
V [Å^3]	5528.5(7)	10682(13)
Z	4	8
$D_{calcd.}$ [gcm^{-3}]	1.237	1.251
μ[mm^{-1}]	0.085	0.085
Reflections	9760	7663
R_{int}	0.0882	0.0705
Parameters	717	760
Restraints	7	292
$R1$ [I > 2σ(I)][a]	0.0561	0.1148
$R1$ (all data)	0.0890	0.1513
$wR2$ [I > 2σ(I)][b]	0.1310	0.2232
$wR2$ (all data)	0.1490	0.2406
GoF (F^2)	1.024	1.166
$\Delta\rho$ [e Å^{-3}]	+0.71 (-0.30)	+0.50 (-0.31)

[a] $R1 = \Sigma\,||F_o| - |F_c||\,/\,\Sigma\,|F_o|$; $R2w = [\Sigma[w(F_o^2 - F_c^2)^2] / \Sigma[w(F_o^2)^2]]^{1/2}$

[b] $w = q[\sigma^2(F_o^2) + (aP)^2 + bP]^{-1}$.

NMR spectra of compounds

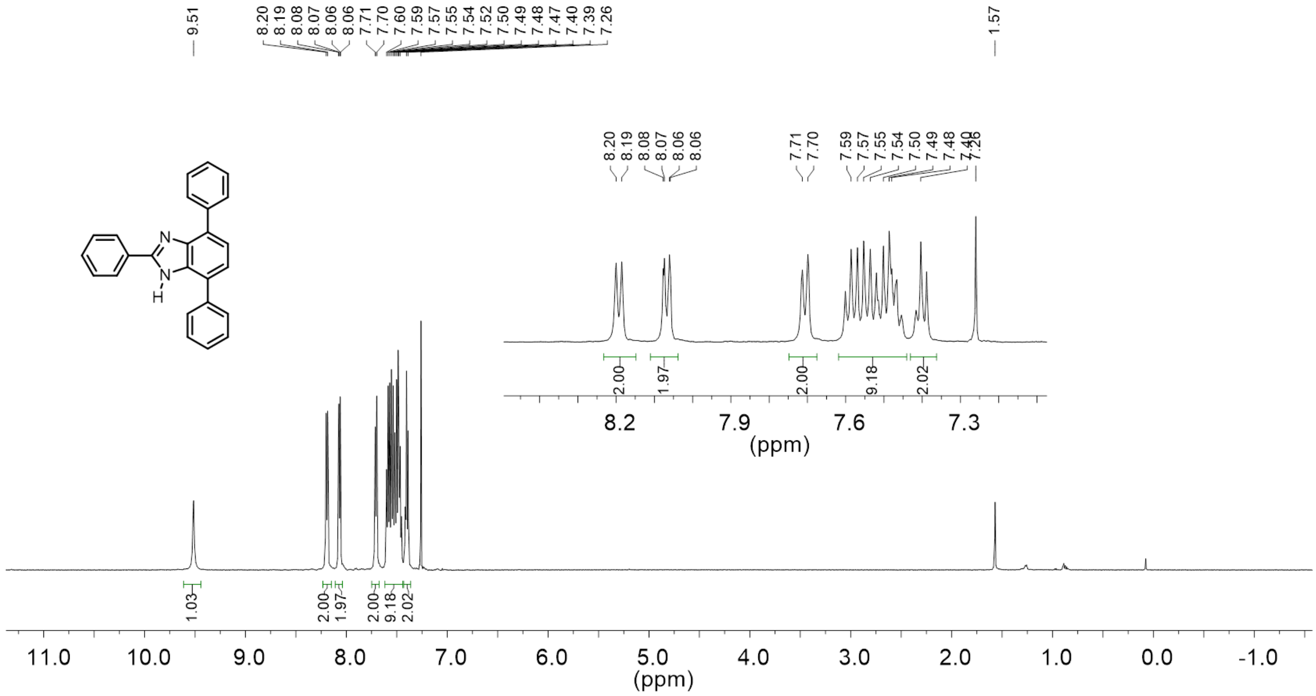

Figure S5 ^{1}H NMR spectrum of **1** in $CDCl_3$

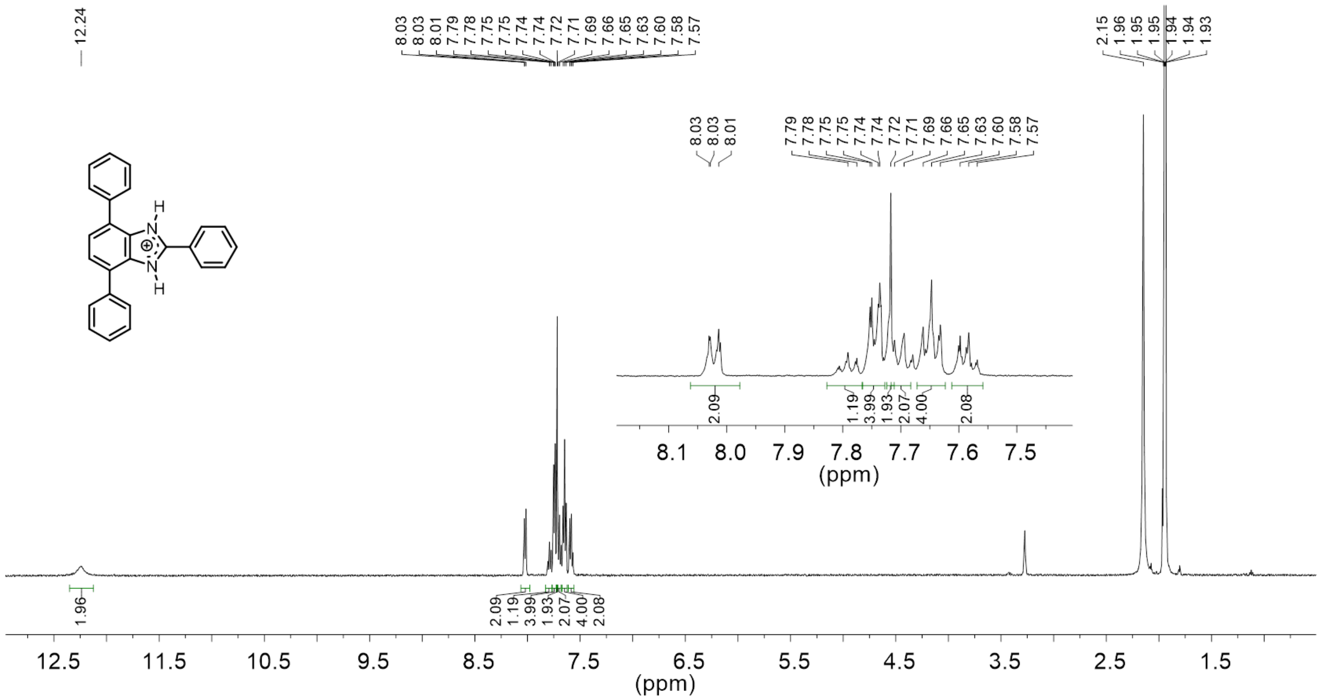

Figure S6 ^{1}H NMR spectrum of [**1**-H][BF_4] in CD_3CN

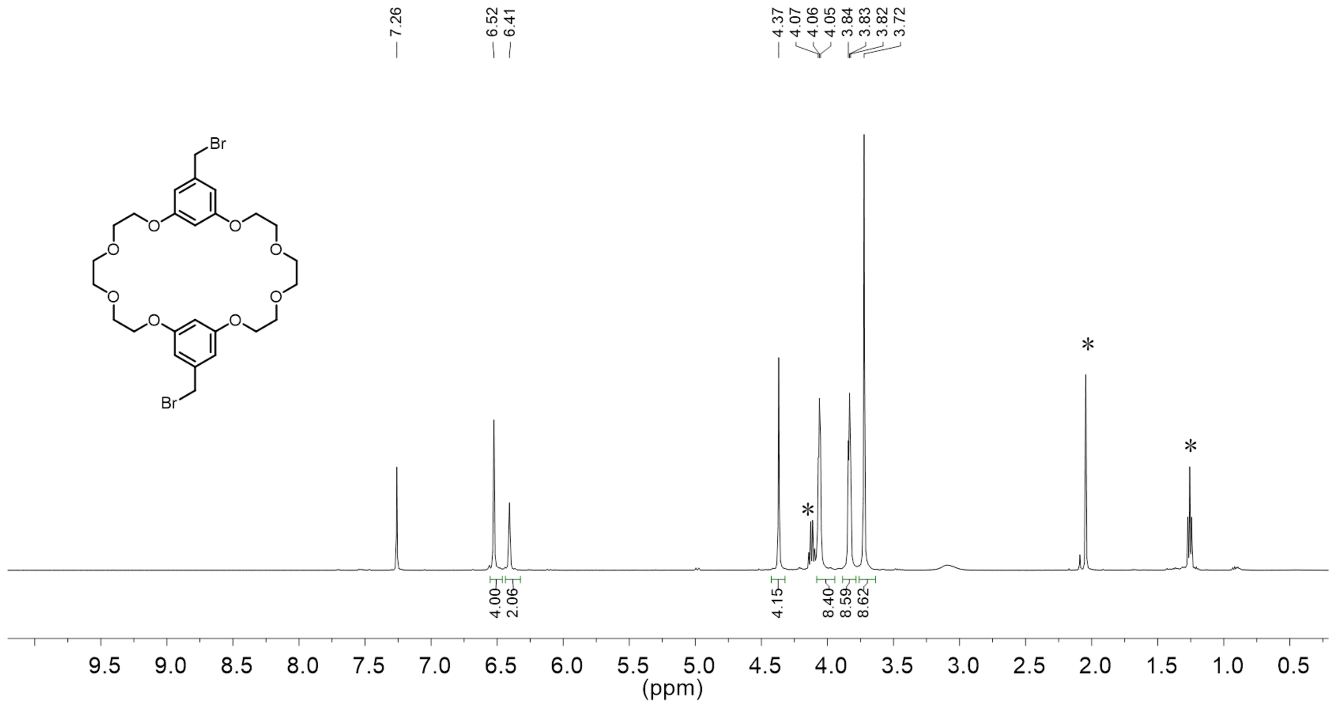

Figure S7. ^{1}H NMR spectrum of **3** in $CDCl_3$ (*:ethyl acetate)

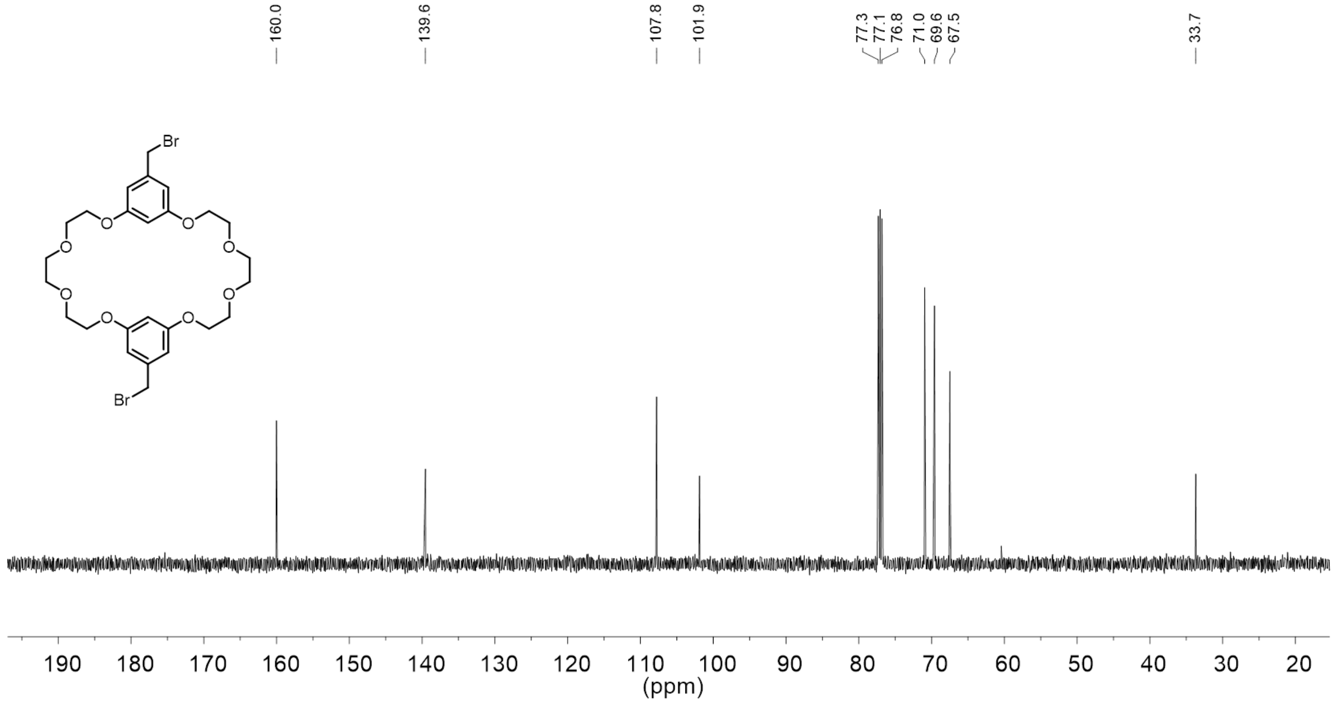

Figure S8. ^{13}C NMR spectrum of **3** in $CDCl_3$

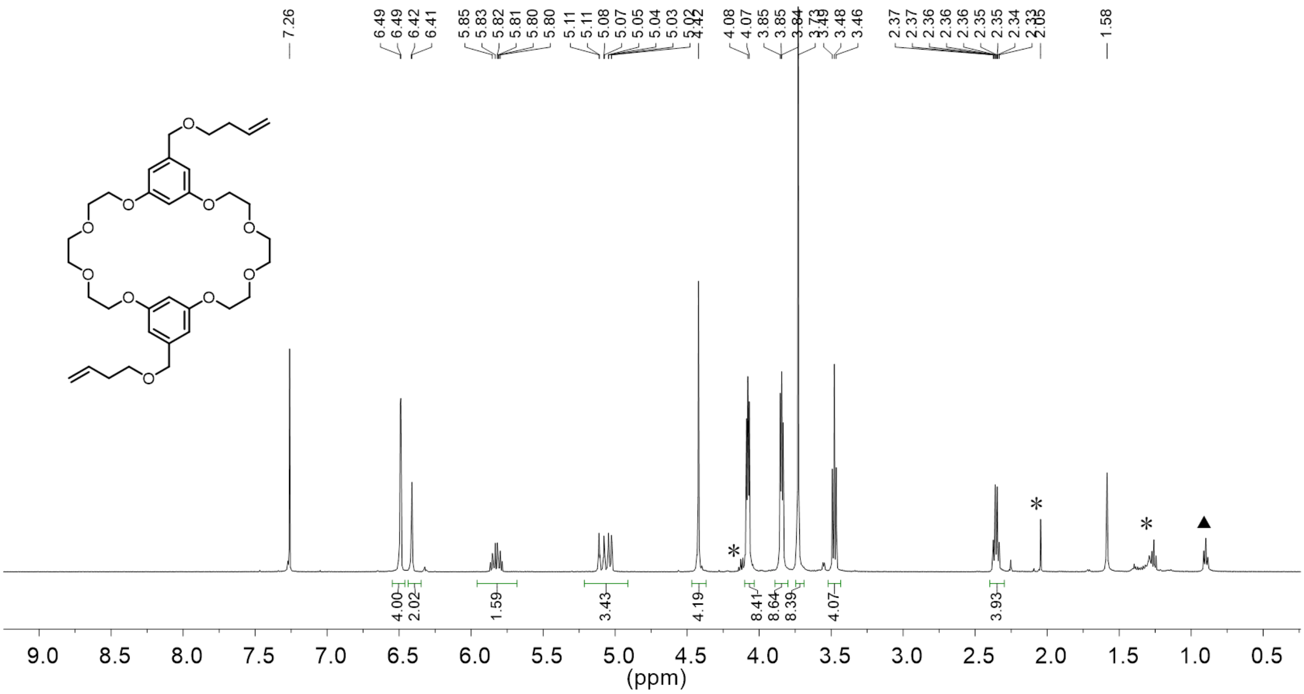

Figure S9. ^{1}H NMR spectrum of **4** in $CDCl_3$ (*:ethyl acetate, ▲:hexane)

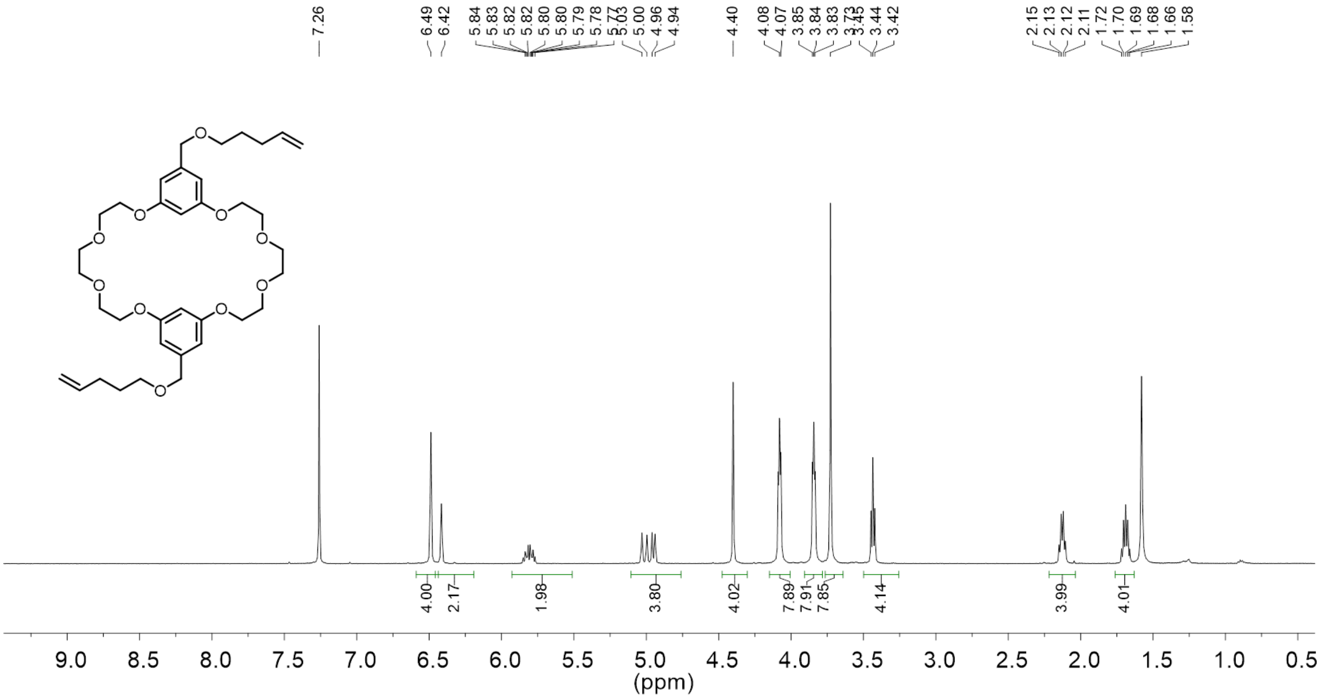

Figure S10. ^{1}H NMR spectrum of **5** in $CDCl_3$

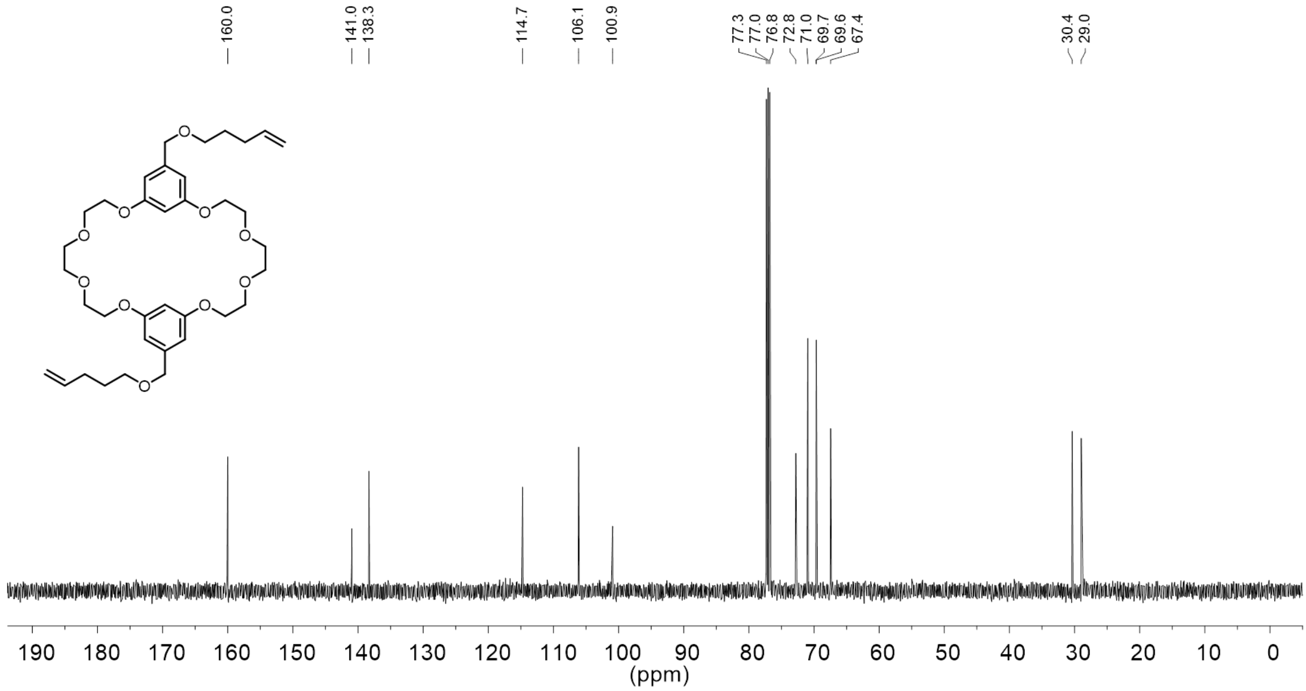

Figure S11. ^{13}C NMR spectrum of **5** in $CDCl_3$

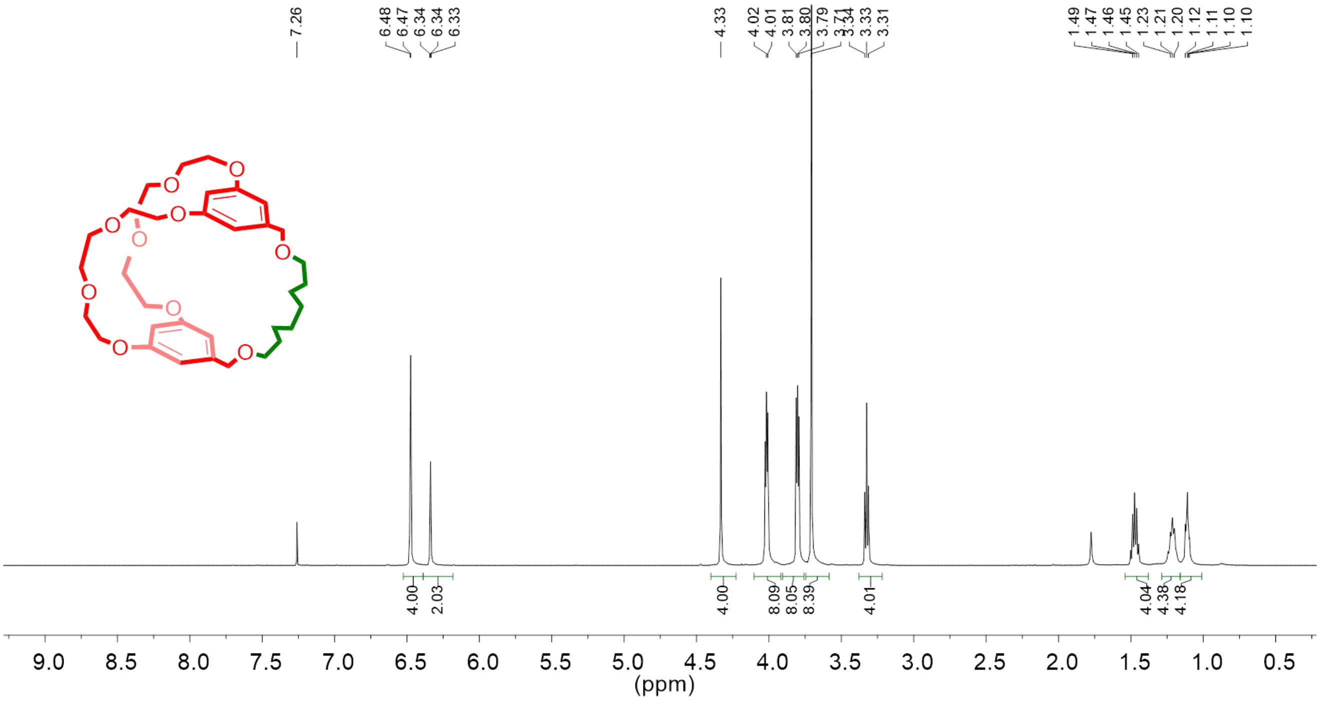

Figure S12. ^{1}H NMR spectrum of **7** in $CDCl_3$

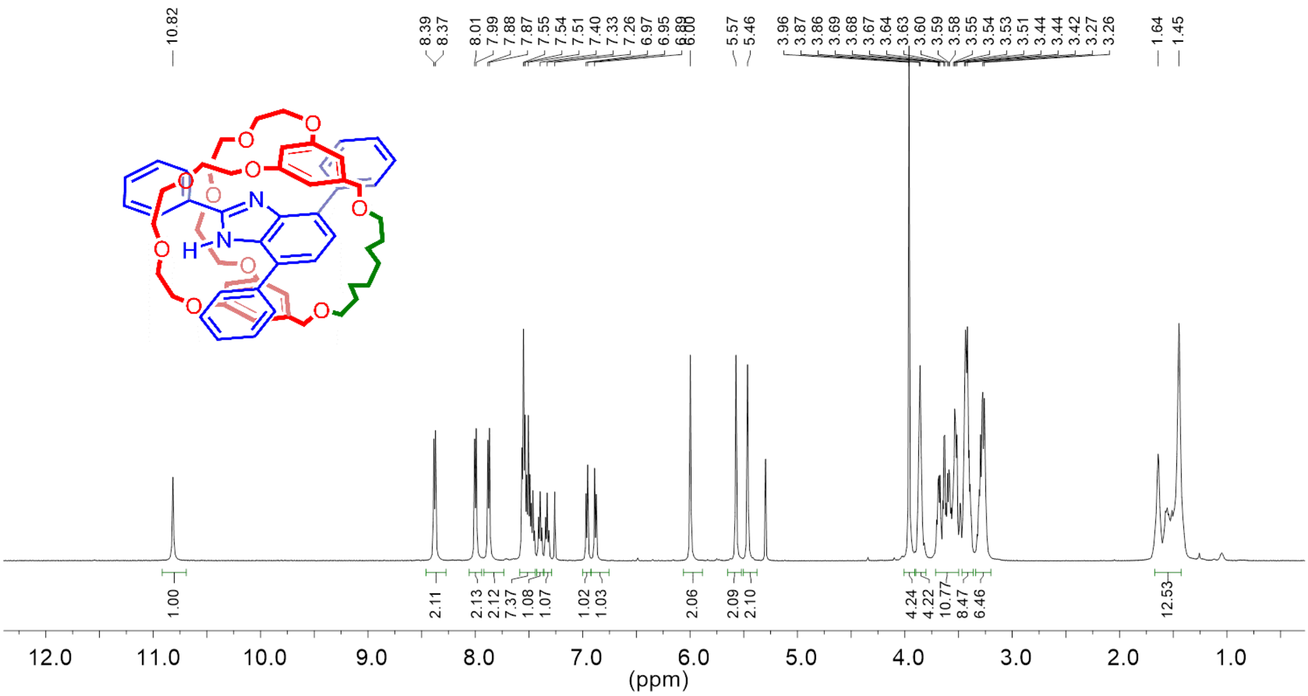

Figure S13. ^{1}H NMR spectrum of **6a** in $CDCl_3$

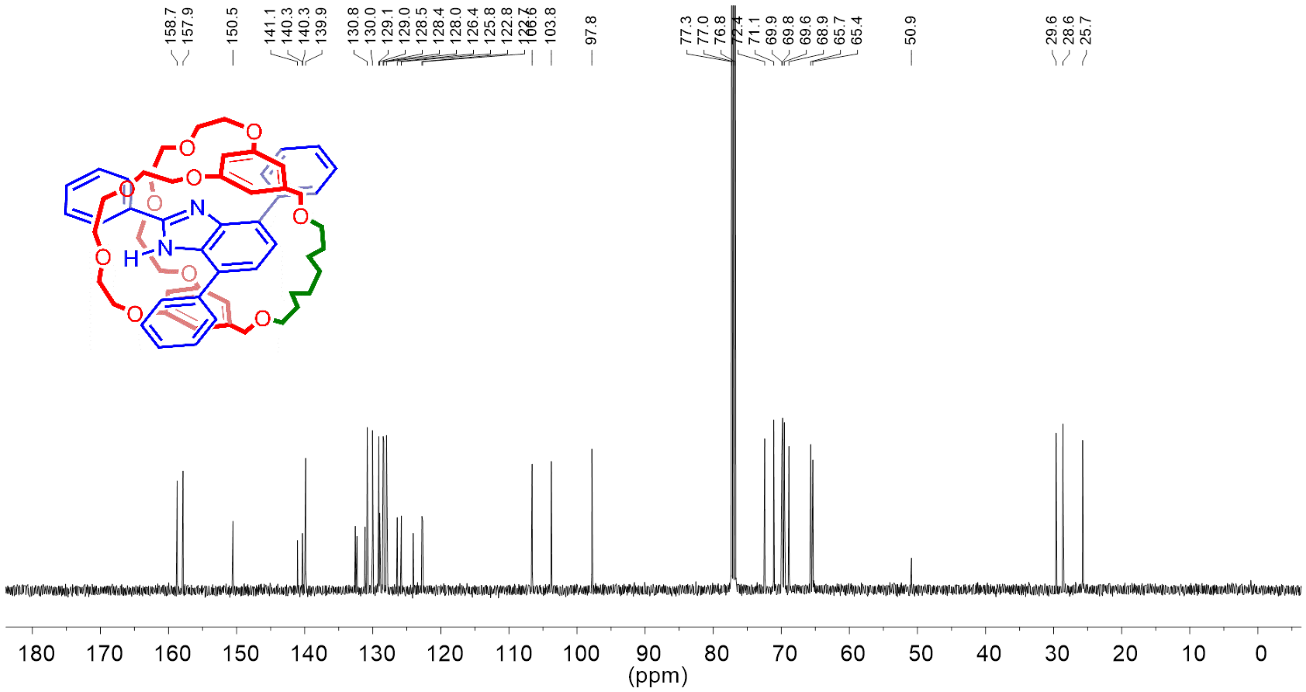

Figure S14. ^{13}C NMR spectrum of **6a** in $CDCl_3$

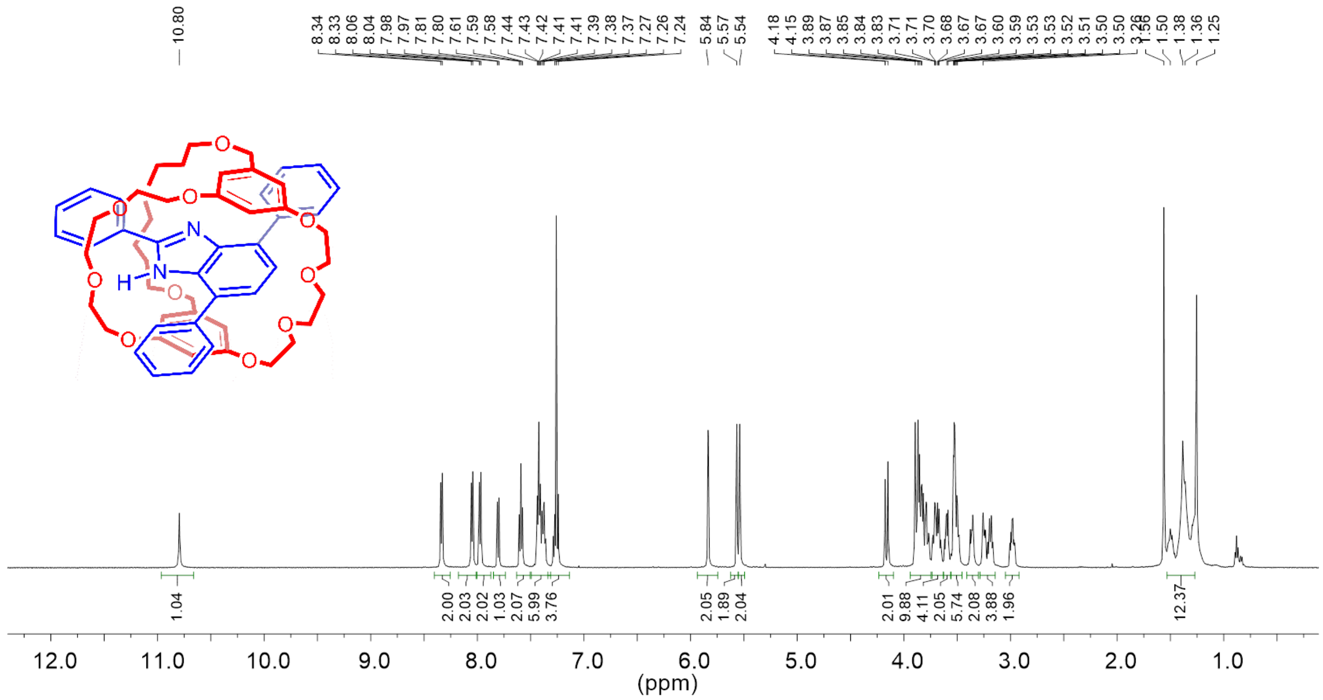

Figure S15. ^{1}H NMR spectrum of **6b** in $CDCl_3$

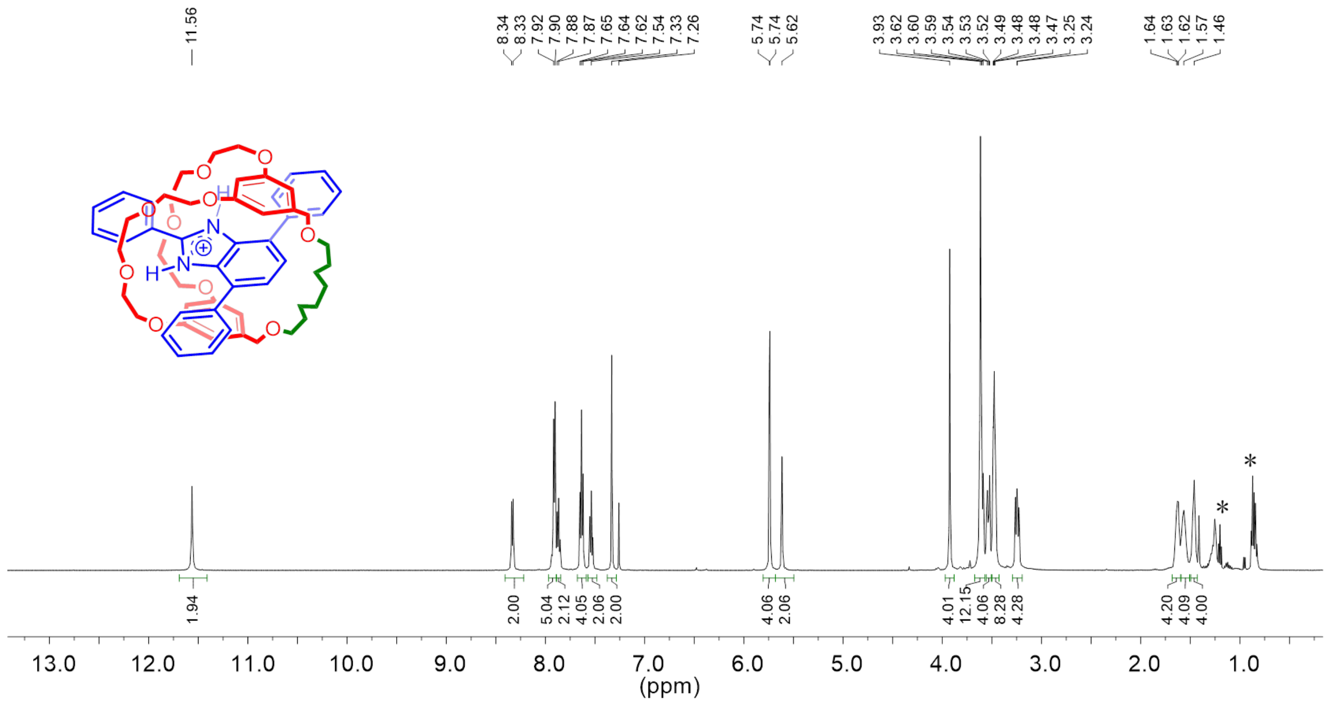

Figure S16. ^{1}H NMR spectrum of [**6a**-H]$^+$ in $CDCl_3$

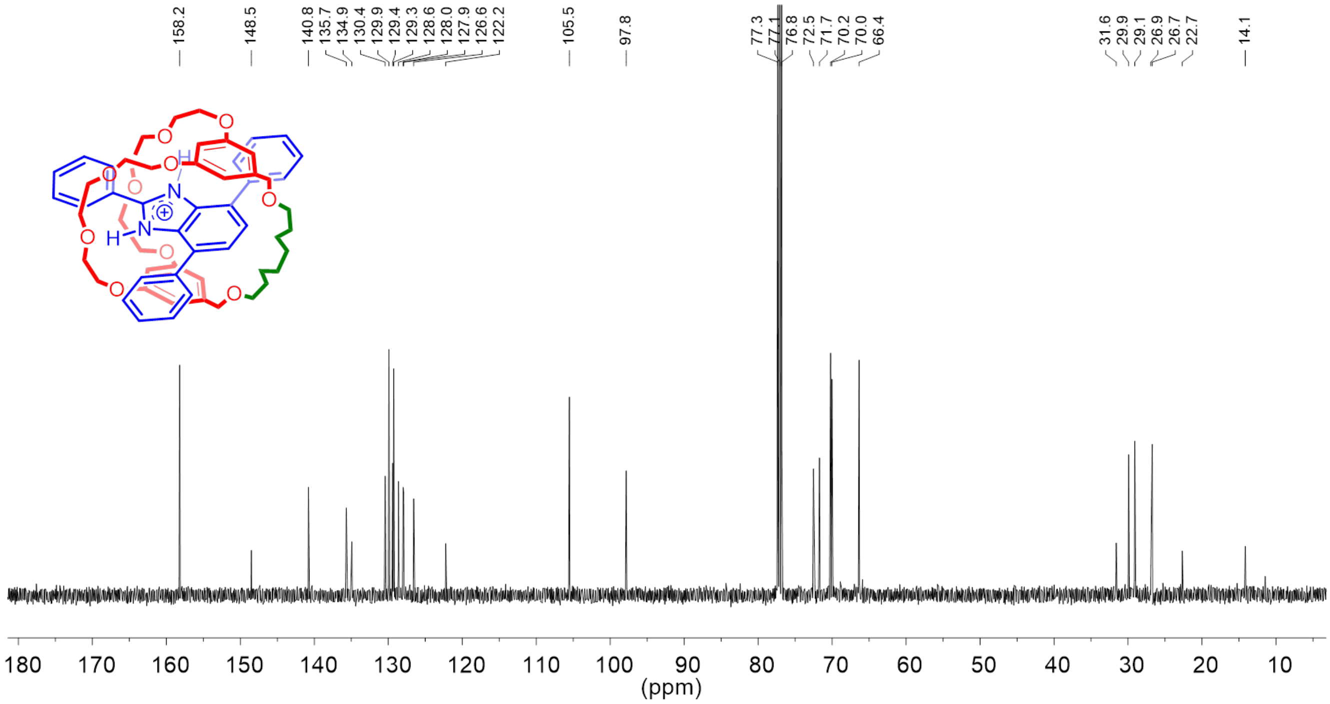

Figure S17. ^{13}C NMR spectrum of **[6a**-H]$^{+}$ in $CDCl_3$

Reference

S1. F. S. Mancilha, B. A. Da Silveira Neto, A. S. Lopes, P. F., Jr. Moreira, F. H. Quina, R. S. Goncalves, J. Dupont, *Eur. J. Org. Chem*. 2006, 4924.

S2. N. Noujeim, K. Zhu, V. N. Vukotic, S. J. Loeb, *Org. Lett*. 2012, **14**, 2484.

S3. F. Wang, Q. Zhou, K. Zhu, S. Li, C. Wang, M. Liu, N. Li, F. R. Fronczek, F. Huang, *Tetrahedron*, 2009, **65**, 1488.

S4. Bruker, (2012). SAINT+ and SADABS. Bruker AXS Inc., Madison, Wisconsin, USA.

S5. G. M. Sheldrick, *Acta Cryst.* 2015, **C71**, 3-8.

S6. O. V. Dolomanov, L. J. Bourhis, R. J. Gildea, J. A. K. Howard, H. Puschmann, *J. Appl. Cryst.*, 2009, **42**, 339-341.
